# Predicting how varying moisture conditions impact the microbiome of dust collected from the International Space Station

**DOI:** 10.1186/s40168-024-01864-3

**Published:** 2024-09-10

**Authors:** Nicholas Nastasi, Ashleigh Bope, Marit E. Meyer, John M. Horack, Karen C. Dannemiller

**Affiliations:** 1 Environmental Science Graduate Program, Ohio State University, Columbus, OH 43210 USA; 2 Department of Civil, College of Engineering, Environmental, and Geodetic Engineering, Ohio State University, 470 Hitchcock Hall, 2050 Neil Ave, Columbus, OH 43210 USA; 3 Division of Environmental Health Sciences, College of Public Health, Ohio State University, Columbus, OH 43210 USA; 4 NASA Glenn Research Center, Cleveland, OH 44135 USA; 5 Department of Mechanical and Aerospace Engineering, College of Engineering and John Glenn College of Public Affairs, Ohio State University, Columbus, OH 43210 USA; 6 Sustainability Institute, The Ohio State University, Columbus, OH 43210 USA

**Keywords:** Built environment, Fungi, Spacecraft, Relative humidity, Microbiome, Space biology, Moisture, Mold

## Abstract

**Background:**

The commercialization of space travel will soon lead to many more people living and working in unique built environments similar to the International Space Station, which is a specialized closed environment that contains its own indoor microbiome. Unintended microbial growth can occur in these environments as in buildings on Earth from elevated moisture, such as from a temporary ventilation system failure. This growth can drive negative health outcomes and degrade building materials. We need a predictive approach for modeling microbial growth in these critical indoor spaces.

**Results:**

Here, we demonstrate that even short exposures to varying elevated relative humidity can facilitate rapid microbial growth and microbial community composition changes in dust from spacecraft. We modeled fungal growth in dust from the International Space Station using the time-of-wetness framework with activation and deactivation limited growth occurring at 85% and 100% relative humidity, respectively. Fungal concentrations ranged from an average of 4.4 × 10^6^ spore equivalents per milligram of dust in original dust with no exposure to relative humidity to up to 2.1 × 10^10^ when exposed to 100% relative humidity for 2 weeks. As relative humidity and time-elevated increased, fungal diversity was significantly reduced for both alpha (*Q* < 0.05) and beta (*R*^2^ = 0.307, *P* = 0.001) diversity metrics. Bacteria were unable to be modeled using the time-of-wetness framework. However, bacterial communities did change based on constant relative humidity incubations for both beta (*R*^2^ = 0.22, *P* = 0.001) and alpha diversity decreasing with increasing moisture starting at 85% relative humidity (*Q* < 0.05).

**Conclusion:**

Our results demonstrate that moisture conditions can be used to develop and predict changes in fungal growth and composition onboard human-occupied spacecraft. This predictive model can be expanded upon to include other spacecraft environmental factors such as microgravity, elevated carbon dioxide conditions, and radiation exposure. Understanding microbial growth in spacecraft can help better protect astronaut health, fortify spacecraft integrity, and promote planetary protection as human activity increases in low-Earth orbit, the moon, Mars, and beyond.

Video Abstract

**Supplementary Information:**

The online version contains supplementary material available at 10.1186/s40168-024-01864-3.

## Background

In the next decade, more people than ever will be working and living in space. This will be fueled in part by the National Aeronautics and Space Agency (NASA) Commercial Low-Earth Orbit Destination initiative that will support private companies such as Starlab Space, LLC, and Blue Origin to build and operate the next generation of space stations [1]. In addition, NASA’s *Artemis* missions will establish a lunar orbiting space station named Gateway, as well as a surface habitat, which will be the proving ground for technologies needed to live on Mars [2]. One of the best analogs we have available to these future systems is the International Space Station (ISS).

The ISS is a unique built environment that is an almost completely closed indoor system continuously inhabited for over 20 years in low-Earth orbit. The ISS contains its own unique microbiome with over 12,000 species identified onboard [3], similar to indoor environments on Earth [4]. These microbes originate from the astronauts, their food, onboard experiments, and other sources [5, 6]. Similar to Earth, many microbes reside in the dust [7].

On Earth, indoor dust acts as a sink [8] for microbes and a potential exposure source [9] for occupants in the indoor environment. Dust is also generated onboard the ISS from the daily activities of the crew. The crew must use a vacuum to remove dust from the high-efficiency particulate air (HEPA) filter coverings, which are a part of the air ventilation system, on a weekly basis [10]. The exposure risk for microbes in dust is not well understood in microgravity environments, but it differs from Earth due to the altered particle size distributions in the air and the absence of gravitational settling influences how they deposit in the lungs when inhaled [11–13].

Exposure to fungal growth indoors is associated with negative health outcomes such as asthma and allergies [14, 15]. Understanding indoor microbial exposure risks is especially critical in the extreme conditions experienced during spaceflight that compromise the human immune system, potentially exacerbating any negative health effects [16]. Additionally, the unique stressors of a spaceflight environment can cause changes in microbial communities such as increased virulence [17] and antimicrobial resistance [5]. Negative health impacts due to microbial exposure have impacted ISS crew members during flight with symptoms such as rhinitis, skin infections, urinary tract infections, and skin rashes [18]. In addition to astronaut health, preventing microbial growth during spaceflight is important to maintain the structural integrity of the spacecraft as some microbes are able to degrade mission-critical spacecraft materials such as plastics [19], sealants [20], and fibers [21] which can cause premature failure of spacecraft components. Microbial activity onboard has been known to cause issues in essential systems such as biofouling in water lines due to issues in the original design that has since been rectified [22]. In addition, capture of bacterial- and fungal-related airborne particles is a major function of the ISS air filtration system [23].

Increased levels of relative humidity facilitate rapid microbial growth, especially for fungi. Elevated equilibrium relative humidity (ERH) in the air is sufficient to provide enough water availability to microbes to support growth in the dust [8, 24, 25], which can be predicted even under varying relative humidity conditions using the time-of-wetness framework [26]. These elevated ERH conditions significantly alter community composition and change microbial function in terrestrial house dust [24, 25, 27]. On the ISS, the current NASA operational guidelines for relative humidity is to maintain it between 25 and 75% [28], but pockets of elevated moisture conditions are possible during the daily operations onboard. Examples include fungal growth on plant experiments onboard due to a ventilation failure that caused high ERH [29], fungal growth on fabric panels where a wet towel was placed [30] on various pieces of equipment used onboard [31], and microbial growth on free-floating condensate on the Russian station *Mir*in an unused section of the station [32]. However, we do not yet understand if we are able to predict fungal growth in dust from the ISS as can be done on Earth.

The goal of this project is to predict fungal growth in dust from the ISS under varying moisture conditions using the time-of-wetness framework. To our knowledge, this is the first study to actively grow a collection of microbes directly from a substrate that is onboard the ISS to model unexpected high moisture conditions. The results have important implications for protecting human health and spacecraft integrity during future missions to the moon, Mars, and beyond.

## Methodology

### Overview

Dust samples were obtained from four vacuum bags that came from weekly housekeeping activities to clean the protective screen covers for the HEPA filters which are part of the ISS air ventilation system. Samples were returned to Earth, where they were incubated at varying constant ERH conditions (50%, 60%, 70%, 80%, 85%, 90%, and 100%) for 2 weeks. In addition, the time-of-wetness framework was used to model microbial growth at elevated (85% ERH) and saturated (100% ERH) conditions. Quantitative polymerase chain reaction (qPCR) was used for the quantification of bacteria and fungi for all samples. Illumina MiSeq sequencing was used to determine how microbial communities in the ISS dust changed for each ERH and time-of-wetness condition. In addition, a subset of these samples was observed via scanning electron microscopy to visualize microbial growth directly on the ISS dust samples.

### ISS dust samples

All dust used in this study was obtained from vacuum bags (CELOC hypo-allergenic filter system Oreck# PKBB12DW) from the vacuum onboard the ISS which the astronauts use to clean the HEPA filter coverings that are part of the air ventilation system. It is important to note that we had no control over when, how much, or where these vacuums were used while onboard, which means other areas may have also been vacuumed in addition to the HEPA filter coverings. In total, ISS dust from four separate vacuum bags was returned to Earth and analyzed in this study. ISS vacuum bags 1–4 were collected by the ISS crew on GMT 264 (September 21, 2018), GMT 102 (April 12, 2019), GMT 243 (August 31, 2019), and GMT 286 (October 13, 2019) and returned to Earth on SpaceX16 (January 13, 2019), SpaceX-17 (July 3, 2019), and SpaceX-19 (January 7, 2020). In total, it took approximately 6, 5, 6, and 4 months for bags 1, 2, 3, and 4 to reach our lab at the Ohio State University from the actual collection date onboard ISS. For ISS vacuum bag 1, the full vacuum bag was sent directly to The Ohio State University Indoor Environmental Quality Laboratory. ISS vacuum bags 2–4 were first sent to the Toxicology and Environmental Chemistry group at NASA’s Johnson Space Center (Houston, TX, USA), and then, a subsample of dust was sent to us for analysis. In addition, we received dust samples that were frozen onboard the ISS, returned in cold stowage, and sent directly to our laboratory, where they were stored at − 80 °C until use (Additional file 1: Table S1).

### Incubations

All incubation was completed in a sterilized 3.8 L glass chamber and placed in a VWR incubator (Model TFFU20F2QWA Radnor, PA USA) set to 25 °C. Sodium chloride (NaCl) and magnesium chloride (MgCl_2_) solutions were used to simulate 50%, 60%, 70%, 80%, 85%, and 90% ERH conditions. Deionized (DI) water alone was used to create 100% ERH conditions. Salt solutions were measured for water activity (*a*_*w*_) using an Aqualab™ Dew Point Water Activity Meter (Decagon 125 Devices Pullman, WA, USA) and adjusted as needed. All incubation chambers contained approximately 50 mL of salt solution or DI for the desired ERH condition and an Onset® HOBO® Data logger (Onset Computer Corporation, Bourne, MA, USA) to monitor ERH and temperature. Dust samples were not sieved and measured out into approximately 25 mg portions that were placed on sterile aluminum foil on a plastic dish. Two-week incubations consisted of triplicate samples for each ISS bag and ERH condition (three samples from each bag per incubation chamber) similar to previous studies [24].

Time-of-wetness incubations followed a procedure previously outlined [25]. Time-of-wetness refers to the time fraction per day when relative humidity conditions are above the 80% threshold [26]. This framework was applied to microbial growth in ISS dust by calculating the relative growth rates at days 5, 10, 14, and 21 at elevated (85% ERH) and saturated (100% ERH) conditions which cycled between 50% ERH for 6, 12, 18, and 24 h during separate incubations. To obtain the relative growth rate (*R*/*k*), we first calculated the growth rates (*k*) for constant (24 h/day) elevated and saturated conditions. Next, the effective growth rates (*R*) were calculated for each condition (elevated and saturated) at 6-, 12-, and 18-h time points. The relative growth rate was calculated for each bag at each time of wetness first, then these values were averaged to obtain the averaged maximum relative growth rate for each time point and ERH condition. Samples were extracted and quantified on days 5, 10, 14, and 21 after incubation (one 25-mg dust sample per day per bag). All incubation chambers were covered with parafilm to retain ERH conditions and allow CO_2_ to escape.

### DNA extractions and qPCR

DNA was extracted from all dust samples using a DNeasy Powerlyzer Power Soil Kit (Qiagen, Hilden, Germany) with a modified bead mixture (1 g garnet, 0.3 g 100 µm glass beads, and 0.1 g 500 µm beads) to allow for more efficient lysis of cells in the dust substrate [33]. Each DNA extraction run included a blank and was confirmed to contain no microbial DNA. A total of 50 µL of DNA extract was collected for each sample and stored at − 20℃ until use.

qPCR was used to determine fungal and bacterial concentrations for all samples with an Applied Biosystems™ QuantStudio™ 6 Flex System (Fisher Scientific, Waltham, MA, USA) using QuantStudio Real-Time PCR Software version 1.3. All samples were diluted to a 50X solution in Tris–EDTA (TE) buffer solution, and triplicate qPCR measurements were made for each sample. For every plate run on qPCR, two wells of template controls were included, and all wells consisted of a 25-µL reaction volume, which included 2 µL of sample.

Total fungal and bacterial quantities in samples were determined by the use of universal assays that measured at the whole kingdom level. SYBR® Green (Applied Biosystems™), forward primer (FF2) 5′-GGTTCTATTTTGTTGGTTTCTA-3′, and reverse primer (FR1) 5′-CTCTCAATCTGTCAATCCTTATT-3′ was used to quantify fungal concentration which targets the 18S rRNA gene [34]. *Aspergillus fumigatus* was grown on Difco™ Potato Dextrose Agar and after 14 days spores were collected, counted, and DNA extracted for use as standards for qPCR fungal quantification. For bacteria, TaqMan® master mix (Applied Biosystems™), forward primer 5′-TCCTACGGGAGGCAGCAGT-3′, reverse prime 5′-GGACTACCAGGGTATCTAATCCTGTT-3′, and PROBE (6-FAM)-5′-CGTATTACCGCGGCTGCTGGCAC-3′-(BHQ) was used to target the 16S rRNA gene [9, 34, 35]. *Bacillus atrophaeus* was grown in Difco™ Luria–Bertani Broth, Miller overnight, counted, and DNA extracted for qPCR bacterial quantification standards. Fungal and bacterial standards were counted using a Labomed microscope with a 20X air objective lens and InCyto DHC-N01-5 Neubauer Improved C-Chips. These standards were run in duplicate for each qPCR plate with a total of six dilutions ranging from 10^1^ to 10^6^ cells or spores/µL. For bacteria, this was reported as cells per milligram of dust, while fungi were reported as spore equivalents per milligram of dust. The term “spore equivalent” refers to the fact fungi are eukaryotic, and the DNA measured with qPCR could be from a spore or from another fungal structure present such as hyphae or different fungal propagules. qPCR cycling parameters for fungal and bacterial primer sets consisted of one cycle of 50℃ for 2 min and 95℃ for 10 min, followed by 40 cycles of 95℃ for 15 s and 60℃ for 1 min. All qPCR values were multiplied by a dilution factor (50X), and the 50 µL extraction volume was then divided by the dust mass of each sample. A subset of samples went through qPCR inhibition testing by spiking *A. fumigatus* and *B. atrophaeus* onto the dust with no inhibition of the reaction detected. The molecular methods used in this study, namely qPCR values are reported in cells/spore equivalents and will not account for differences in DNA extraction efficiency, amplification bias, or gene copy number between species [36, 37].

### Microbial sequencing

All sequencing for dust samples was performed on an Illumina MiSeq™ at RTL Genomics (Lubbock, TX, USA). All samples were sequenced for fungi, and a smaller subset was sequenced for bacteria. For fungal sequencing, ITS1F (CTTGGTCATTTAGAGGAAGTAA) and ITS2aR (GCTGCGTTCTTCATCGATGC) ribosomal DNA primers were used [38], while the bacteria used 515F (5′-GTGCCAGCMGCCGCGGTA) and 806R (5′-GGACTACHVHHHTWTCTAAT) primers [39] with 2 × 300 bp sequencing reads.

The Quantitative Insights Into Microbial Ecology 2 (QIIME2), version 2021.8, bioinformatics pipeline was used to analyze raw FASTQ sequencing data [40]. For fungi, primers and spacers were trimmed using the Cutadapt [41] plug-in and paired-ends joined using the VSEARCH join-pairs method [42]. Sequences were then trimmed to a Phred score of 30 with three low-quality base windows using the Quality-filter plugin [43]. Fungal sequences were clustered using the vsearch open reference method with UNITE database reference sequences (version 9.0) [44]. Bacterial sequences were run through the Dada2 pipeline [45] and clustered by phylogeny [46]. Beta diversity metrics for fungi were analyzed using Bray–Curtis dissimilarity while bacteria utilized unweighted and weighted UniFrac statistics with a principle coordinate analysis (PCoA) and Adonis function in QIIME2 [47]. Richness and Shannon alpha diversity metrics were calculated for all incubations and significance determined using the Kruskal–Wallis test statistic from QIIME2.

The Basic Local Alignment Search Tool (BLAST) version 2.9.0 [48], the User-friendly Nordic Internal Transcribed spacer Ectomycorrhiza (UNITE) 2019 database [49], and Fungal High-throughput Taxonomic tool for use with Next-Generation Sequencing (FHitINGS) [50] version 1.4 was used to identify fungal taxonomy. A note that an updated 2022 version of the UNITE database now exists, and we compared relative abundance data to our analyses of the 2019 database to which no significant differences for our samples were observed. Bacterial taxa were classified in QIIME2 using the feature-classifier plugin [51] which utilized the Greengenes database version 13_8 [52]. All sequencing data has been submitted to NASA’s GeneLab database (GLDS-623) [53].

### Microscopy and total organic carbon

A subset of dust samples from ISS bag 1 were observed using scanning electron microscopy. Approximately 1.25 mg of dust was placed on black double-sided carbon tape and attached to a SEM sample holder. These samples were then incubated in triplicates for several ERH conditions (50%, 85%, and 100%) for 2 weeks at 25 °C. After incubation, samples were coated with 25 nm of gold and placed in a Apreo™ LoVac Scanning Electron Microscope (Thermo Scientific™ Waltham, MA, USA) for visual observation. Microscopy preparation and analysis were completed at the Center for Electron Microscopy and Analysis (Ohio State University Columbus, OH, USA). In addition, we measured the total soluble organic carbon in the original dust and dust after incubation after 2 weeks at 85% and 100% ERH. A total of four samples were measured at each condition which included one from each bag. Each dust sample was placed into 35 mL of DI in a 50-mL falcon tube. The tubes were placed on a shaker table at 180 rpm for 30 min. After shaking, 30 mL of the solution was extracted and run through a 0.45-µm filter which was then used for analyses. Soluble organic carbon was measured on a Shimadzu TOC-V CSN (VELP Scientific, Inc. Deer Park, NY, USA).

### Statistical analyses

Microbial quantities for constant ERH incubations at each ERH condition tested were compared to quantities in the original dust (no incubation) samples using Satterthwaite two-sample *t* tests and a Spearman rank correlation coefficient utilizing STATA (Version 16.1). Statistical significance was considered (*P*< 0.05). QIIME2 version 2021.8.0 was used to create a principal coordinate analysis (PCoA) plot using Bray–Curtis and UniFrac distance matrices. The Adonis function was also used to compare ERH and TOW conditions for microbial species beta diversity while Kruskal–Wallis was used to determine significance for alpha diversity. The PCoA plot was imported into R Studio (version 2021.09.0 Build 351) for visualization. Statistical Analysis System® Studio, version 9.4, was used to compare taxonomic diversity between ERH conditions, time-of-wetness, frozen, and original dust samples. Species that did not occur in at least 10% of all samples were removed before analysis. All relative abundance data was transformed using the inverse hyperbolic sine function and combined with qPCR quantities to produce an absolute abundance value as previously described [25]. Taxonomy among samples was analyzed using the PROC MULTTEST FDR Test Mean function in SAS. False discovery rate was used instead of positive false discovery rate due to the relatively small sample size [54].

## Results

### Increased relative humidity conditions lead to increased microbial growth

**Fig. 1:**
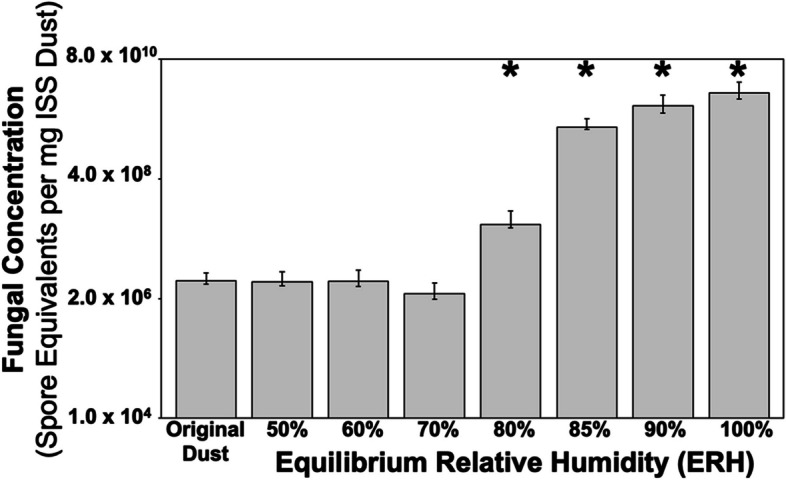
Fungal growth concentrations of original dust and after constant equilibrium relative humidity incubations at 50, 60, 70, 80, 85, 90, and 100% ERH at 25℃. Each fungal concentration represents the average value of four vacuum bags which included physical triplicates for each bag and triplicate qPCR concentrations for each sample for a total of 36 measurements per condition. Error bars represent the standard error of fungal quantities for each vacuum bag. * indicate statistically significant growth (*P* < 0.05) compared to the original dust using Satterthwaite two-sample *t* test

Dust was received from four vacuum bags from the ISS with an average initial fungal concentration of 4.39 × 10^6^ spore equivalents (SE)/mg dust and an average bacterial load of 1.06 × 10^7^ cells/mg dust. After incubating the dust samples for 2 weeks at different ERH conditions to simulate an unexpected high moisture event, the fungal concentration ranged from 4.22 × 10^6^ SE/mg dust at 50% ERH to 2.10 × 10^10^ SE/mg dust at 100% ERH (Fig. 1, Table S2). Increased fungal concentration was associated with elevated ERH conditions after 2 weeks (Spearman rank correlation (*r*_*s*_) = 0.77, *P* < 0.0001) with significantly more fungal growth at 80% (*P* < 0.044) and 85–100% ERH (*P* < 0.0001) (Table S3). Bacterial concentration was significantly increased compared to the original load at 90% (*P* = 0.005) and 100% ERH (*P* = 0.0001) with a positive association between bacterial concentration and ERH (*r*_*s*_ = 0.39, *P* = 0.0001) (Additional file 2: Figure S1A, Table S2–S3). Dust was also collected by tweezers from the ISS HEPA filters and returned frozen for analysis with lower fungal and bacterial loads compared to bulk dust samples (Additional file 1: Table S4). These results are similar to previous studies that showed significant growth under similar conditions for fungi [8, 24] and bacteria [8] in dust collected from Earth-based residential homes. The overall quantity of fungal growth is also comparable between these studies where house dust [24] and ISS dust could both reach more than 10^9^ spore equivalents per milligram of dust when exposed to 100% ERH for 2 weeks.

**Fig. 2:**
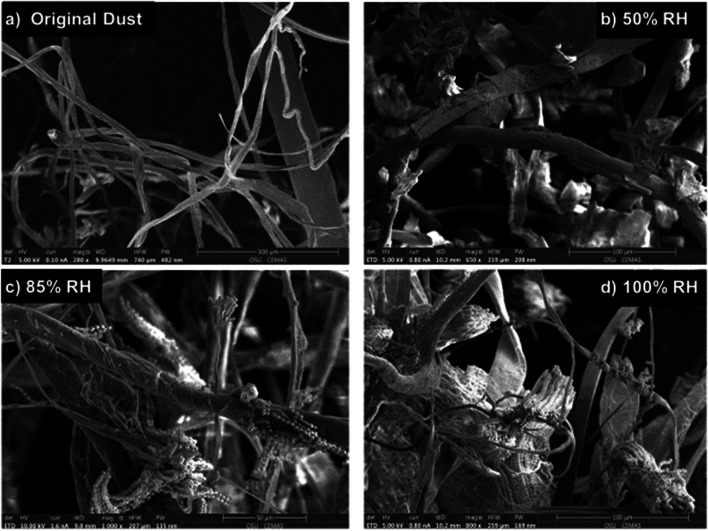
Scanning electron microscopy (SEM) images of ISS dust from the original dust (**A**) and incubations at 50% ERH (**B**) show fibrous dust materials, but no fungal growth. Fungal growth including spores, elongated hyphae, and different fungal propagules was observed in ISS dust incubated at 85% ERH (**C**) and 100% ERH (**D**) for 2 weeks at 25℃

Original dust samples (no incubation or ERH exposure) and dust incubated at 50% ERH for 2 weeks showed no visible signs of microbial growth, in scanning electron microscopy images (Fig. 2A,B). At elevated relative humidity conditions of 85% and 100% ERH, we observed indications of active fungal growth on the dust substrate (Fig. 2C,D). This included elongated hyphae with conidia production prevalent on almost all dust surfaces, which indicates that fungal reproduction is occurring. In general, more of all of these structures were observed at 100% compared to 85% ERH, similar to other studies with Earth dust and carpet samples [24]. Our previous microscopy analyses in residential carpets also concluded that almost no fungal growth occurred even at high ERH conditions if house dust was not present, indicating the nutrients in dust are essential for active growth [24].

**Fig. 3:**
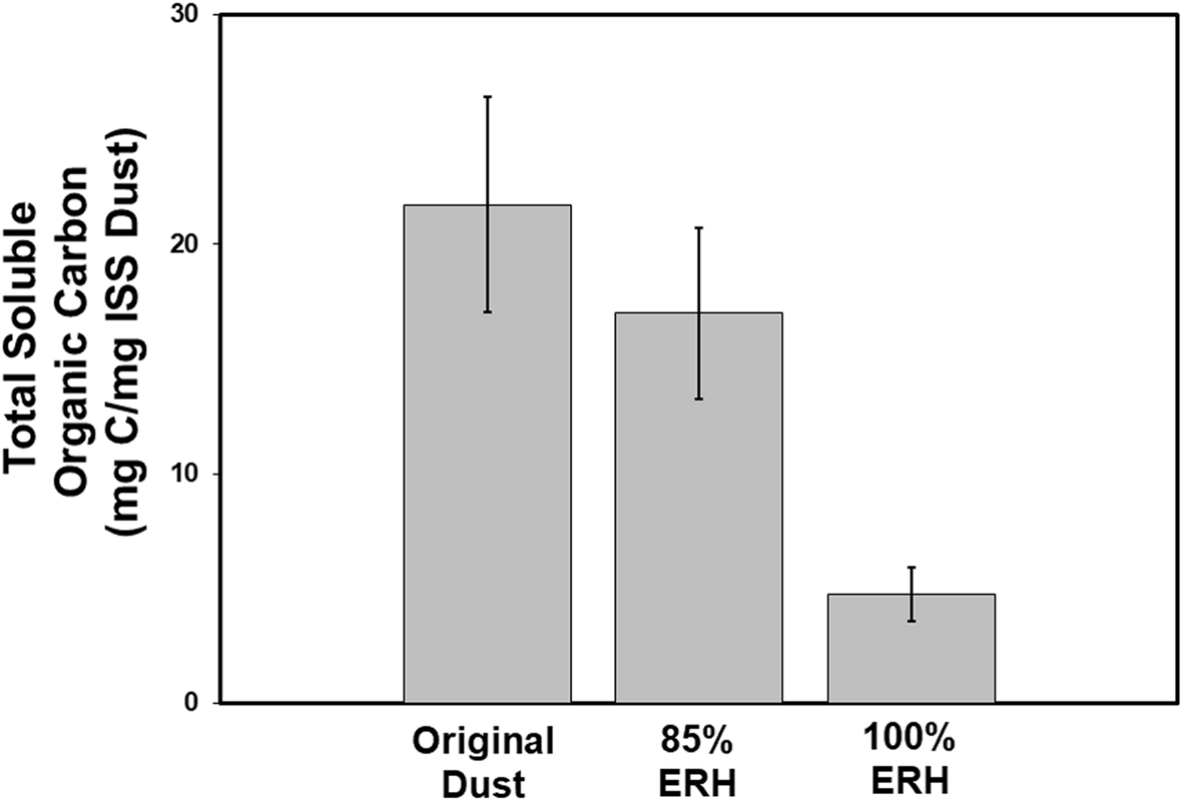
Total soluble organic carbon (TOC) measured in ISS dust in the original dust as well as in dust incubated (25°C) for 2 weeks at 85% and 100% relative humidity conditions. Significant reductions in TOC were observed for 100% conditions but not 85% compared to original dust samples indicating potential fungal use of TOC in ISS dust. Each condition contained four samples (one from each ISS bag in this study), and error bars are the standard deviation of these four bags. * represents a statistically significant (*P* < 0.05) reduction in total soluble organic carbon compared to original dust samples using Satterthwaite two-sample *t* test

Total soluble organic carbon was measured in ISS dust samples that included the original dust as well as dust incubated at 85% and 100% ERH for 2 weeks at 25℃ (Fig. 3). For each condition, a total of four samples were measured that included one sample per ISS bag collected. In the original dust, total soluble organic carbon ranged from 12.55 to 36.79 mg/L with no significant changes after a 2-week incubation at 85% ERH (10.2–29.05 mg/L, *P* = 0.52). In contrast, there was a significant reduction in soluble organic carbon after 2-week incubations at 100% ERH (1.76–8.08 mg/L, *P*= 0.048). This is a soluble organic carbon utilization rate of approximately 1.2 and 0.3 mg soluble carbon per day for 100% and 85% ERH conditions, respectively. From the original ISS dust samples, we measured an average of 22 mg soluble organic carbon per milligram of dust. In comparison, house dust from Earth-based residential homes contains 35 mg of total soluble organic carbon per mg of dust [8]. This indicates that dust from both the ISS and homes on Earth contains significantly more carbon than is required for fungal growth (~ 0.0072 mg soluble carbon/mg dust). Therefore, moisture continues to be the limiting factor for growth.

### Fungal growth predictions vary based on maximum ERH

**Fig. 4:**
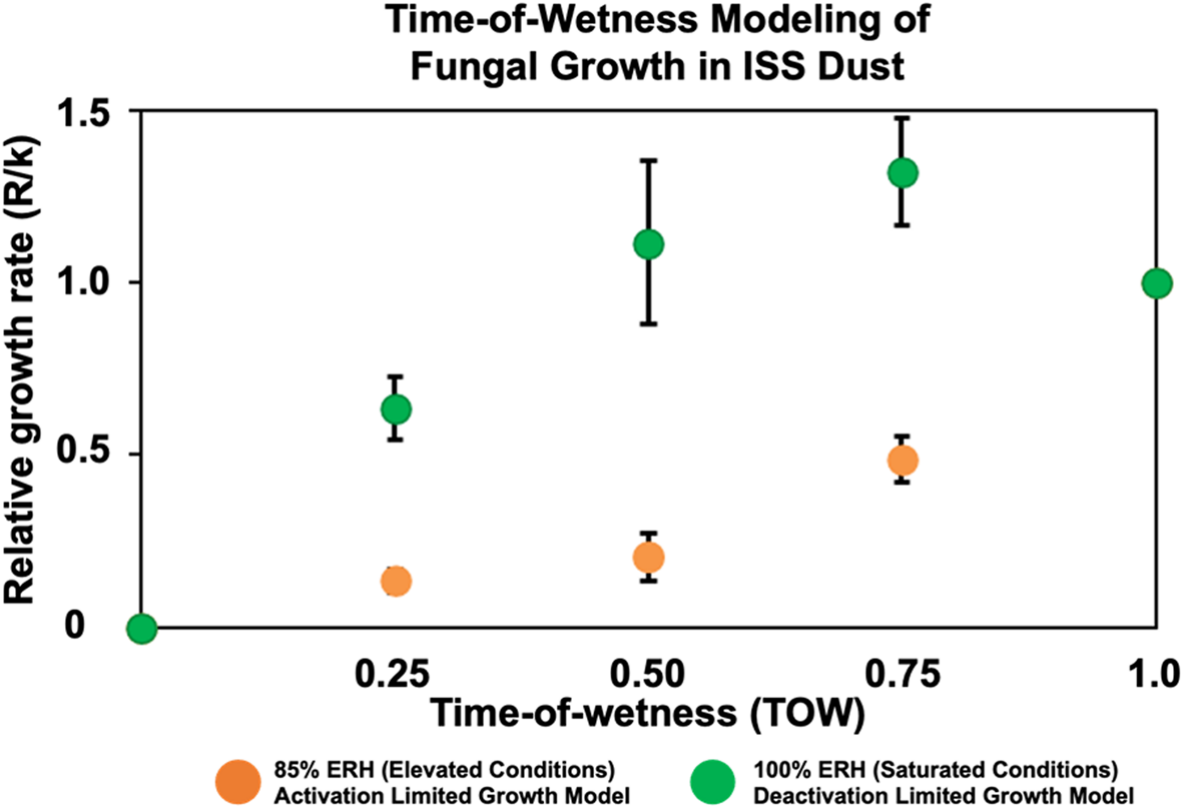
Fungal time-of-wetness modeling for elevated (85% ERH) and saturated (100% ERH) conditions. Elevated conditions (orange) show an activation-limited growth model, while saturated conditions (green) show a deactivation-limited growth model. Error bars shown represent the standard error between all four ISS bags sampled

Relative humidity often fluctuates in indoor spaces, so we applied the time-of-wetness framework to model fungal growth measured under varying ERH conditions (high moisture for 0, 6, 12, 18, and 24 h per day). We observed an activation-limited growth model for elevated (85% ERH) conditions, while at saturated (100% ERH), we observed a deactivation-limited growth model (Fig. 4). At both elevated and saturated conditions, growth was associated with increasing time per day at higher moisture levels (*r*_*s*_ = 0.68, *P* = 0.015). Bacterial growth showed no significant difference between elevated and saturated conditions and did not align with any time-of-wetness growth model (Additional file 2: Figure S1B). On Earth, fungi in house dust samples differed and showed a two-stage activation limited growth model for both elevated (85% ERH) and saturated (100% ERH) conditions, and bacteria also had insufficient growth to apply a model [25]. Effective growth constants and total fungal growth rates for the model are available in Additional file 1: Tables S5–S9. The differences in fungal growth models for Earth and ISS-built environments may be due to different microbial and chemical compositions of the dust because even Earth-based buildings in close proximity can differ. These differences can be attributed to many environmental factors such as the occupants inside, cleaning habits, building materials, temperature, humidity, and other factors.

### Microbial diversity changes as ERH conditions are altered

We analyzed the fungal and bacterial communities in the samples via DNA amplicon sequencing (Table S10). There was a total of 11,180,519 quality fungal reads and 2,502,228 quality bacterial reads present in all sequenced samples. A total of 107 fungal species and 127 bacterial species were present in at least 10% of samples. The most common fungal genera were *Aspergillus* (100% of samples), *Penicilium* (95%), and *Rhodotorula* (94%). The most common fungal species in all samples included *Aspergillus sydowii* (100%), *Aspergillus unguis* (100%), and *Penicillium chrysogenum* (90%). For bacteria, *Bacillales* (99%), *Actinomycetales* (99%), and *Clostridiales* (96%) were the most common order and the most common bacterial species were *Corynebacterium kroppenstedtii* (81%), *Staphylococcus pettenkoferi* (78%), and *Lactobacillus helveticus* (74%).

**Fig. 5:**
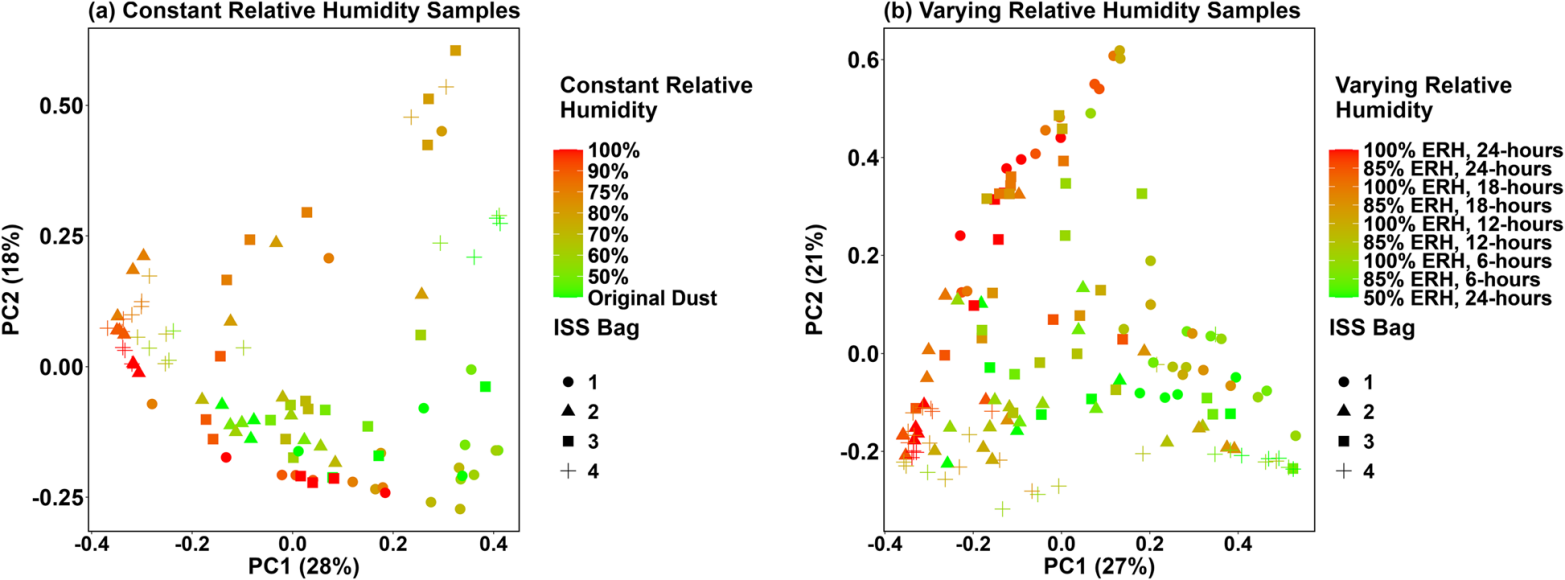
Principal coordinate analyses (PCoAs) of beta diversity fungal species present in ISS dust after 2-week incubations at varying ERH conditions (**A**) and time-of-wetness conditions (**B**). Both scenarios show as ERH increases (green to red) the fungal species composition is altered

After incubation at constant ERH, fungal community composition differed by each bag (*R*^2^ = 0.233, *P* = 0.001) and ERH condition (*R*^2^ = 0.307, *P* = 0.001) (Fig. 5A). In all varying ERH samples, fungal community composition also differed by bag (*R*^2^ = 0.098, *P* = 0.001) and varying ERH conditions (*R*^2^ = 0.090, *P* = 0.001) (Fig. 5B), though to a lesser degree compared to the constant ERH incubation. These differences were more pronounced when limiting the analysis to a single maximum ERH level or time elevated. For example, considering the 24-h incubations only for elevated and saturated conditions shows a more distinct difference in species (*R*^2^ = 0.201, *P* = 0.001). PCoA plots for all conditions is shown in Additional file 2: Figures S2–S4 and Additional file 1: Table S11). Bacterial beta diversity analyses showed similar trends of community composition changing with increased ERH and time-of-wetness conditions (Additional file 2: Figure S5, Additional file 1: Table S12). Finally, frozen dust samples were compared to the original dust and constant ERH incubation samples at 50, 85, and 100% ERH. For both fungi and bacteria, frozen samples clustered close to the original dust and 50% ERH samples but were more dissimilar to 85 and 100% ERH samples (Additional file 1: Figure S6, Additional file 1: Table S13).

**Fig. 6:**
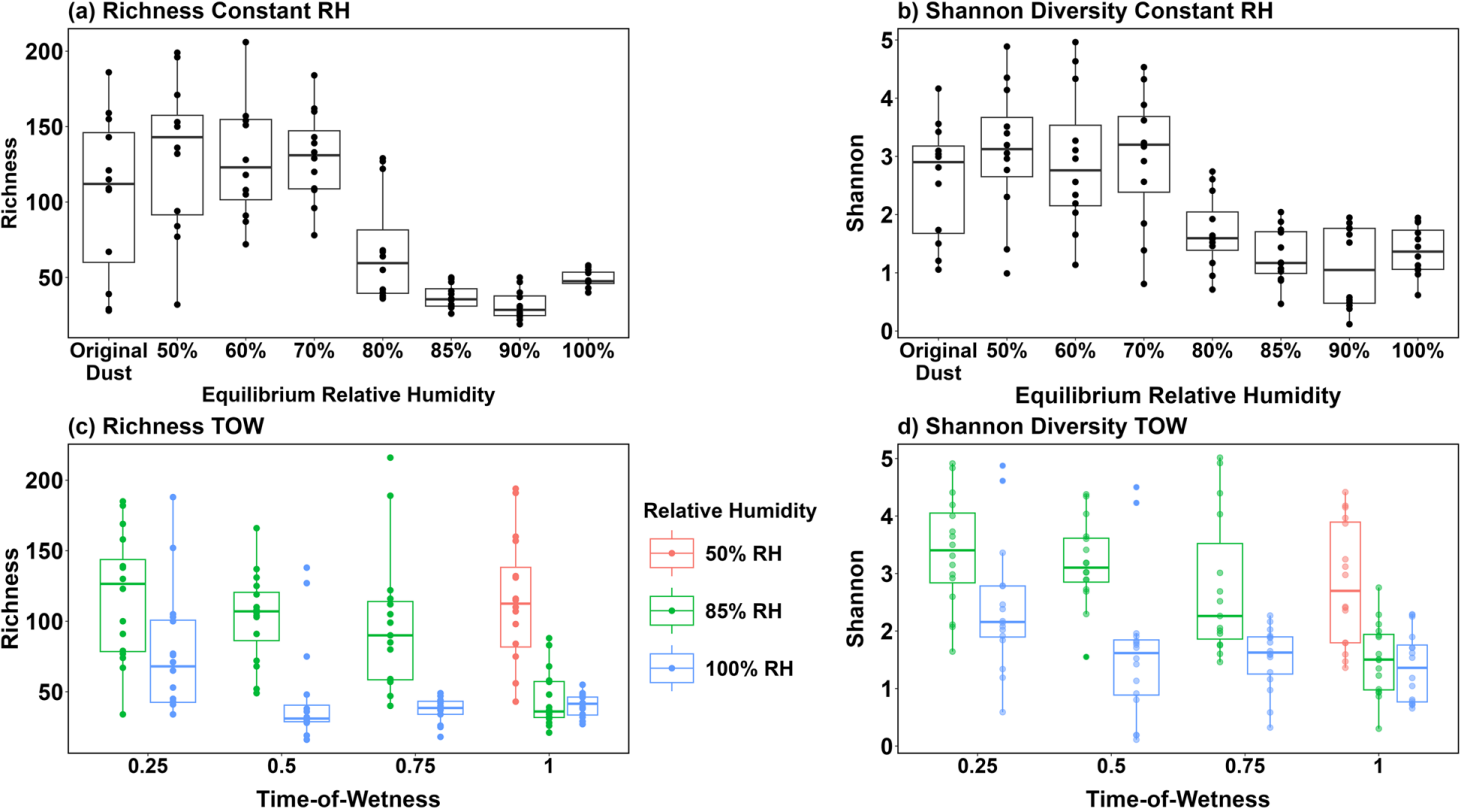
Fungal alpha diversity plots for constant ERH, 2-week incubations (**A**, **B**), and time of wetness incubations (**C**, **D**). For constant incubations, the *x*-axis represents the original dust and ERH conditions after 2 weeks of incubation (**A**, **B**). Time-of-wetness incubations show the time elevated on the *x*-axis as well as the ERH conditions tested (50% = red, 85% = green, and 100% = blue). Note that 50% of ERH conditions for time-of-wetness samples were only performed at a time elevated of one (24 h per day)

Moisture also impacted sample diversity. For fungal constant ERH incubations, richness was significantly decreased (*Q* < 0.05) compared to original dust samples starting at 85% ERH, while Shannon diversity differences appeared at 80% ERH (Fig. 6A, B, Additional file 1: Table S14). For time-of-wetness samples at saturated (100% ERH) conditions, there were significant changes in both fungal richness and Shannon diversity at extended times elevated (12, 18, and 24 h) compared to the shorter times tested (0 and 6 h) (Fig. 6C,D). We also limited analyses to specific ERH conditions and times elevated (Additional file 1: Table S15). Bacterial samples also showed significant changes in richness and Shannon diversity during constant ERH incubations at 85%, 90%, and 100% ERH compared to original dust samples, while time-of-wetness samples also showed significant differences between 50 and 100% ERH conditions for 24 h (Additional file 1: Figure S7A, Additional file 1: Table S16). Finally, frozen samples were compared to original dust samples as well as samples from constant ERH incubations at 50%, 85%, and 100% ERH. For bacteria, there was a significant difference in both richness and Shannon diversity for all conditions tested; however, fungi only showed these differences at 85% and 100% ERH for 2 weeks compared to the frozen dust samples (Additional file 1: Figure S7B, Additional file 1: Table S17).

Differential abundance analyses were used to compare fungal species between all incubation conditions in this study. Comparing fungal species at non-elevated (original dust, 50, 60, 70% ERH) to elevated (80, 85, 90, 100% ERH) conditions identified a total of 77 species more abundant at non-elevated conditions compared to only six species more abundant at elevated conditions (Additional file 1: Table S18). This indicates that at elevated ERH conditions (> 80% ERH) fungi such as *Aspergillus sydowii* (*P* < 0.0001), *Aspergillus unguis* (*P* < 0.0001), *Aspergillus nidulans* (*P* < 0.0001), *Aspergiullus subversicolor* (*P* < 0.0001), *Penicillium chrysogenum* (*P* = 0.0016), and *Aspegillus hongkongensis* (*P* = 0.0071) may be growing, significantly reducing fungal diversity in the ISS dust. Under varying relative humidity conditions, there were generally more species associated with lower moisture conditions (Additional file 1: Tables S19–S21). These analyses were also performed for bacteria and found no significant differences in bacterial species between any of the incubation times and ERH conditions tested (Additional file 1: Table S23).

**Fig. 7:**
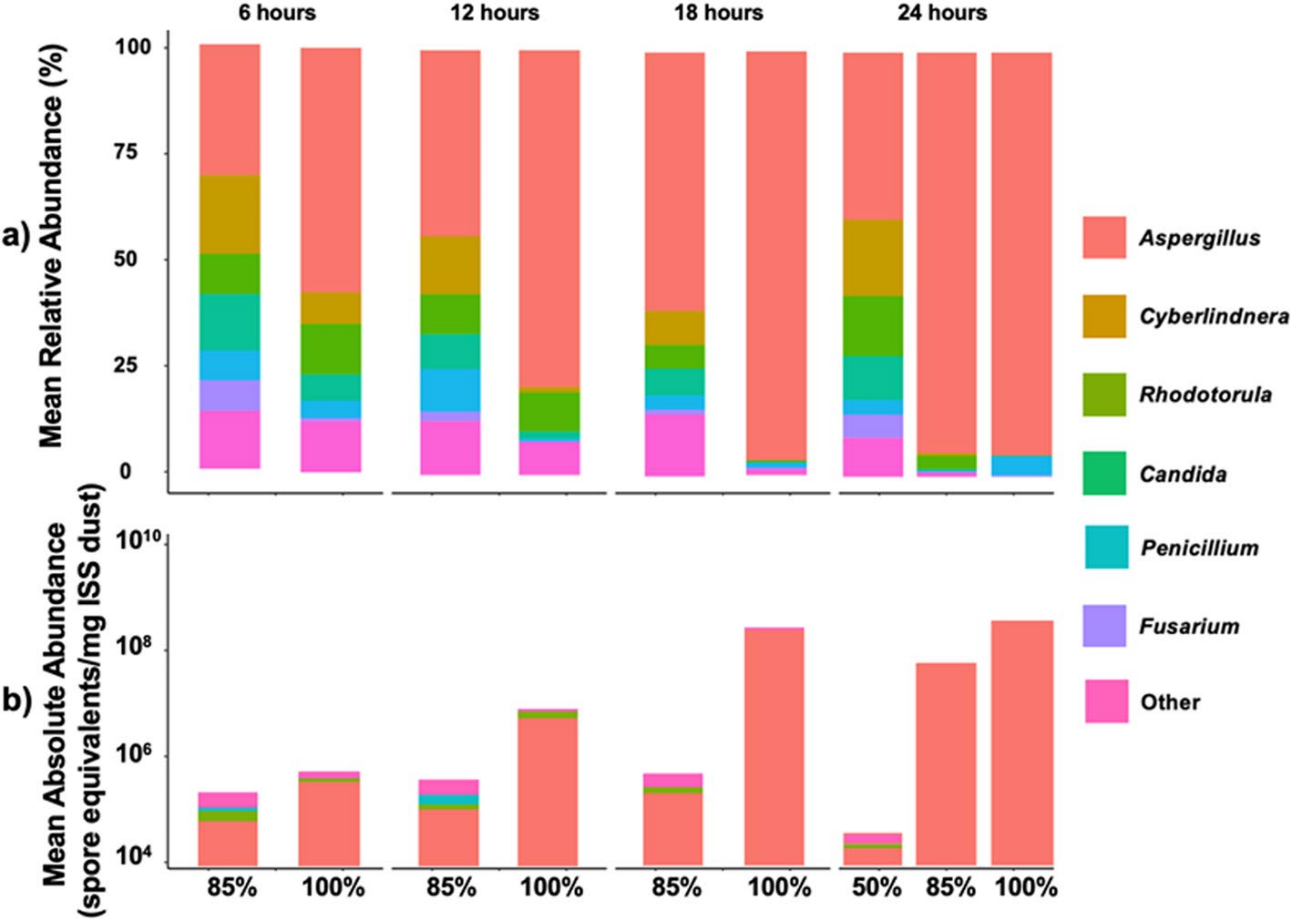
Mean relative (**A**) and absolute (**B**) abundance data for the fungal genus of time-of-wetness samples. Abundances represent elevated (85%) and saturated (100%) relative humidity conditions at 6-, 12-, 18-, and 24-h time-of-wetness samples as well as unmodified (50%) at 24 h. The mean abundances here represent the average for day 5, 10, 14, and 21 dust samples for each relative humidity and time-of-wetness condition for the four total ISS vacuum bags tested in this study (a total of 12 samples for each ERH/time-of-wetness condition)

*Aspergillus* is the dominant genus, and it becomes more dominant as ERH and time elevated increases (Fig. 7). A similar trend is also shown in the constant ERH incubations (Additional file 1: Figure S8), where *Aspergillus* is the most dominant at 85% and 90% ERH. Interestingly, at 100%, *Penicillium* starts to grow significantly more compared to other elevated ERH conditions (80%, 85%, and 90% ERH). Finally, we compared original dust samples (no incubation or ERH exposure) to frozen dust samples (Additional file 1: Figure S9). For relative abundance, the original dust samples contained more *Aspergillus*, *Cyberlinda*, and *Alternaria*, while the frozen samples appeared to contain more *Rhodotorula*, *Malassezia*, and *Rhodosporidiobolus*. There was also a significant difference in absolute abundance for all fungal genera with up to 10^4^ spore eq/mg dust present in the original dust samples compared to the frozen samples.

## Discussion

To date, all microbial studies that have been performed related to spaceflight samples have focused on characterizing what is there including water, surface, dust, and air samples [55–57], but not what happens when moisture becomes elevated and unintended growth occurs. To our knowledge, this is the first study to expose ISS dust to varying moisture conditions to show that increasing ERH alters microbial growth and community composition even for a short period of time. As mentioned above, NASA sets standards for ERH conditions onboard the ISS that ideally stay between 25 and 75% ERH [28]. However, due to the airflow present in the ISS ventilation system, pockets of isolated moisture can occur in certain spots around the station especially during certain activities. For example, astronauts are required to exercise for 2.5 h daily to mitigate the many negative health impacts associated with long-duration spaceflight [58] which creates excess moisture in the form of sweat. Personal hygiene is also a major source of potential elevated moisture exposure while showering, brushing teeth, or washing hands. Additionally, this moisture may originate from the crew dining area during meals or from unintended water release from several plant production systems onboard [59, 60]. In microgravity fluids such as water act differently compared to on Earth, such as capillary forces being more dominant on the ISS (microgravity) where Earth is governed by gravitational forces. Due to these many potential sources of daily elevated moisture conditions, it is critical to understand how fungal and bacterial communities in spacecraft dust will react when receiving ideal growth conditions, namely water availability, and how this can affect crew health, spacecraft integrity, and planetary protection plans.

In ISS dust after 2 weeks exposed to constant ERH conditions significant growth for fungi occurred at 80% RH, while for bacteria this occurred at 90% ERH compared to original dust samples (no ERH exposure). This is similar to previous studies that showed significant growth under similar conditions for fungi [5, 8] and bacteria [5] in dust collected from Earth-based residential homes. The overall quantity of fungal growth is also comparable between these studies where house dust [8] and ISS dust could both reach more than 10^9^SE per milligram dust when exposed to 100% RH for 2 weeks. The time-of-wetness framework has also been applied to growth in Earth-based house dust samples that can be compared to samples from ISS dust. Time-of-wetness samples showed a two-stage activation limited growth model for fungi for both elevated (85% RH) and saturated (100% RH) conditions [9]. These results differ in contrast to the ISS dust where elevated RH fungi showed an activation-limited growth model and at saturated conditions showed a deactivation-limited growth model (Fig. 4). There may be several potential reasons for this difference in fungal growth models for Earth and ISS built environments. Generally, every indoor environment has a different microbial dust composition. For future missions, especially beyond low-Earth orbit, understanding the microbes present and how they may grow will be essential to mitigate any potential negative impacts on crew health and safety due to microbial-related illness or material degradation.

These results offer a path to improved prediction of fungal growth in specialized indoor spaces modeled on fluctuating ERH conditions. However, it is important to restate that these incubations were performed on Earth and not in environmental conditions experienced in low-Earth orbit such as microgravity, elevated CO_2_, and low-dose radiation, all of which may affect microbial growth and microbial composition. The results are also affected by the dust sampling protocol for returned vacuum bags, which were triple-sealed and stored at room temperature while awaiting return to Earth in which time microbial communities may have been altered. Given the difficulty of procuring spaceflight samples, we have a relatively small sample size (dust from four ISS vacuum bags), but this should be sufficient to provide valuable information. These limitations highlight the need for future on-orbit studies to better understand this microbiology during actual spaceflight conditions.

## Conclusion

Our results provide important foundational knowledge for the prediction of fungal growth in future spacecraft, space stations, and surface habitats. The time-of-wetness framework, which utilizes varying ERH conditions, as shown in this study can be expanded and refined to incorporate other key environmental factors present in spaceflight. With the appropriate model based on time exposure to elevated relative humidity levels, we can predict how much and what type of microbial growth will be present in spacecraft dust during long-duration human space missions. We can continue to refine moisture control on spacecraft to preserve a healthy environment. As crewed commercial spacecraft and missions beyond low-Earth orbit become a reality, a better understanding of microbes onboard a spacecraft will protect human health and spacecraft integrity and aid in developing planetary protection protocols to prevent forward and/or backward microbial contamination of Earth and the other celestial bodies we will visit in the future [61].

## Supplementary Information

Additional file 1: Table S1: Frozen dust sample dates and location. These frozen samples were not vacuumed, instead they were picked from the location and placed into a sterile bag. They were then frozen at -80C until use in this study. Table S2: Fungal and bacterial concentrations for 2-week incubation samples at 25℃ and each ERH condition tested. Table S3: Summary of Satterthwaite two-sample t-test statistics for fungal and bacterial 2-week incubations. Table S4: qPCR values for fungal and bacterial quantities for frozen dust sample and the original dust collected from the ISS vacuum bags. Table S5: Total fungal growth rates for TOW incubations. Values represent the average of the 4 ISS bags collected. Table S6: qPCR values for all TOW samples. Table S7: Effective growth rate constants (k) for TOW at constant (24 h per day) ERH conditions. Table S8: Effective growth constants (R) for all TOW samples. Table S9: Relative growth constants (R/k) for all TOW samples. Table S10: Most common taxa that was present in all sequenced samples sorted by order, genus, and species for bacteria and fungi. Table S11: Adonis values for fungal bray Curtis PCoA analysis for each time-of-wetness condition. Table 12: Adonis statistics for bacterial time of wetness beta diversity measurements. Table S13: Adonis statistics for fungal and bacterial frozen sample comparisons. Frozen samples were compared to original dust samples (from ISS vacuum bag) as well as 2-week incubations at 50%, 85%, and 100% RH. Table S14: Fungal alpha diversity Kruskal–Wallis statistics for richness and Shannon diversity for 2-week incubations at each RH conditions tested. Significant changes in both richness and Shannon diversity compared to the original dust began to occur at 80% RH (Q < 0.05). Table S15: Richness and Shannon diversity Kruskal–Wallis statistics for fungal time-of-wetness samples. Table S16: Kruskal–Wallis test statistics for alpha diversity metrics for all sequenced bacterial samples. Table S17: Kruskal–Wallis test statistics for fungal and bacterial frozen sample comparisons. Table S18: Differential abundance fungal comparison between non-elevated (Original Dust, 50, 60, 70% RH) and elevated (80, 85, 90, 100% RH) after 2-week constant ERH incubations at 25℃. Table S19: Differential abundance fungal comparison between original dust samples and 24-h TOW saturated (100% RH) conditions. There were 52 fungal species more abundant in the original dust compared to 6 species more abundant at saturated conditions. Table S20: Differential abundance fungal comparison between unmodified (50% RH) 24-h TOW samples and 24-h TOW saturated (100% RH) conditions. There were 29 fungal species more abundant in the unmodified condition compared to 10 species more abundant at saturated conditions. Table S21: Differential abundance fungal comparison between high (85% RH) 24-h TOW samples and 24-h TOW saturated (100% RH) conditions. There was 1 fungal species more abundant in the high condition compared to 8 species more abundant at saturated conditions. Table S22: Differential abundance fungal comparison between high (85% RH) for all TOW samples and all TOW saturated (100% RH) conditions. There were 30 fungal species more abundant in the high condition compared to 4 species more abundant at saturated conditions. Table 23: Example of bacterial differential abundance analysis for non-elevated (original dust and 50% RH) and elevated (80, 85, 90, and 100% RH) conditions for 2-week incubations. No bacterial species were found to be more abundant in either condition. This was true for all time-of-wetness incubation comparisons as well (not shown). Additional file 1: Figure S1: A) Bacterial concentration of original dust and at each ERH condition tested (50, 60, 70, 80, 85, 90, and 100%) after two weeks at 25℃. Quantities for each condition represent a total of 36 qPCR measurements from 4 vacuum bags with triplicate physical samples from each bag and triplicate qPCR measurements per sample. B) Time-of-Wetness models for bacterial growth in ISS dust for elevated (85% ERH) and saturated (100% ERH) conditions. Figure S2: Fungal principal coordinate analyses of time-of-wetness samples separated out by elevated (A) and saturated (B) relative humidity conditions. Figure S3: Fungal principal coordinate analyses of time-of-wetness samples separated out by time points of 6 h (A), 12 h (B), 18 h (C), and 24 h (D). Figure S4: Fungal principal coordinate analyses of 50% ERH time-of-wetness samples separated out by sample days. Figure S5: A) Bacterial PCoA plots for constant ERH 2-week incubations. Only original dust, 50%, 80%, 85%, 90%, and 100% ERH samples were sequenced. B) Bacterial PCoA plot for Time-of-Wetness incubations. Only constant 24-h samples for 50% and 100% ERH conditions were performed for days 5, 10, 14, and 21. Both figures represent weighted unifrac distance matrices. Figure S6: Principal coordinate analyses of frozen dust sample returned from the ISS. Frozen samples were compared to original dust samples (from ISS vacuum bag) as well as 2-week incubations at 50%, 85%, and 100% ERH. Fungi PCoA plots used the Bray–Curtis dissimilarity statistics (C), while bacteria used both weighted (A) and unweighted unifrac (B). Figure S7: Frozen sample alpha diversity plots for (A) bacteria and (B) fungi. Frozen dust samples were compared to original dust, 50% ERH 2-week, 85% ERH 2-week, and 100% ERH 2-week incubations. Figure S8: Mean relative (A) and absolute (B) abundance data for fungal genus in constant equilibrium relative humidity (ERH) incubation samples. Original dust was not incubated and represents what was in the dust in the ISS vacuum bags with no ERH exposure. For each ERH condition, samples were incubated for 2 weeks at 25°C. Each condition (including original dust) represents the mean of 12 total dust samples which includes 3 physical triplicates from the 4 ISS vacuum bags used in this study. Figure S9: Mean relative (A) and absolute (B) abundance data for fungal genus of frozen ISS dust and original dust samples. Original dust was not incubated and represents what was in the dust in the ISS vacuum bags with no ERH exposure. Frozen dust samples were collected onboard the ISS via a tweezer (no vacuum), placed in a triple-sealed plastic bag, and stored at -80°C until use in this study. The abundance data represents of a total of 12 samples for original dust samples (3 for each ISS bag) and 8 frozen dust samples (1 for each location sampled).

## Data Availability

The sequencing data that supports the findings of this study are available in the NASA Open Science Data Repository (OSDR), also known as GeneLab, with identifiers 10.26030/87vb-2280, OSD-694, GLDS-623. All microbial quantification data used in this study can be found in the supplementary information.

## Abbreviations

NASA: National Aeronautics and Space Agency
ISS: International Space Station
HEPA: High-Efficiency Particulate Air
ERH: Equilibrium relative humidity
SE: Spore equivalents
qPCR: Quantitative polymerase chain reaction
PCoA: Principal coordinate analysis
