# Supplementary material for: Predicting how varying moisture conditions impact the microbiome of dust collected from the International Space Station
Source: Microbiome. 2024 Sep 10;12:171. doi: 10.1186/s40168-024-01864-3 (PMC11386075; doi:10.1186/s40168-024-01864-3)
Supplement: Supplementary file 2 — Additional file 1: Table S1: Frozen dust sample dates and location. These frozen samples were not vacuumed, instead they were picked from the location and placed into a sterile bag. They were then frozen at -80C until use in this study. Table S2: Fungal and bacterial concentrations for 2-week incubation samples at 25℃ and each ERH condition tested. Table S3: Summary of Satterthwaite two-sample t-test statistics for fungal and bacterial 2-week incubations. Table S4: qPCR values for fungal and bacterial quantities for frozen dust sample and the original dust collected from the ISS vacuum bags. Table S5: Total fungal growth rates for TOW incubations. Values represent the average of the 4 ISS bags collected. Table S6: qPCR values for all TOW samples. Table S7: Effective growth rate constants (k) for TOW at constant (24 h per day) ERH conditions. Table S8: Effective growth constants (R) for all TOW samples. Table S9: Relative growth constants (R/k) for all TOW samples. Table S10: Most common taxa that was present in all sequenced samples sorted by order, genus, and species for bacteria and fungi. Table S11: Adonis values for fungal bray Curtis PCoA analysis for each time-of-wetness condition. Table 12: Adonis statistics for bacterial time of wetness beta diversity measurements. Table S13: Adonis statistics for fungal and bacterial frozen sample comparisons. Frozen samples were compared to original dust samples (from ISS vacuum bag) as well as 2-week incubations at 50%, 85%, and 100% RH. Table S14: Fungal alpha diversity Kruskal–Wallis statistics for richness and Shannon diversity for 2-week incubations at each RH conditions tested. Significant changes in both richness and Shannon diversity compared to the original dust began to occur at 80% RH (Q < 0.05). Table S15: Richness and Shannon diversity Kruskal–Wallis statistics for fungal time-of-wetness samples. Table S16: Kruskal–Wallis test statistics for alpha diversity metrics for all sequenced bacterial samples. Table S1 [file 40168_2024_1864_MOESM1_ESM.pdf]

Supplementary Information

Predicting how varying moisture conditions impact the microbiome of dust collected from the International Space Station

Nicholas Nastasi[1,2,3], Ashleigh Bope[1,2,3], Marit E. Meyer[4], John M. Horack[5], and Karen C. Dannemiller[2,3*].

[1]Environmental Science Graduate Program, Ohio State University, Columbus, OH 43210
[2]Department of Civil, Environmental, & Geodetic Engineering, College of Engineering, Ohio State University, Columbus, OH 43210
[3]Division of Environmental Health Sciences, College of Public Health, Ohio State University, Columbus, OH 43210
[4]NASA Glenn Research Center, Cleveland, OH 44135
[5]Department of Mechanical and Aerospace Engineering, College of Engineering and John Glenn College of Public Affairs, Ohio State University, Columbus, OH 43210
*Corresponding Author

Supplemental Table S1: Frozen dust sample dates and location. These frozen samples were not vacuumed, instead they were picked from the location and placed into a sterile bag. They were then frozen at -80□C until use in this study.

Sample date	Sample Location
GMT 111	NOD3 D3-01 HEPA
GMT 111	LAB15D5
GMT 111	NOD1D301
GMT 118	LAB101 IMV INLET
GMT 118	LABS5D HEPA
GMT 118	NOD3D2-15 INLET
GMT 118	NOD1D3 01 HEPA GRATE INLET
GMT 118	NOD3D3-01 HEPA INLET GRATE

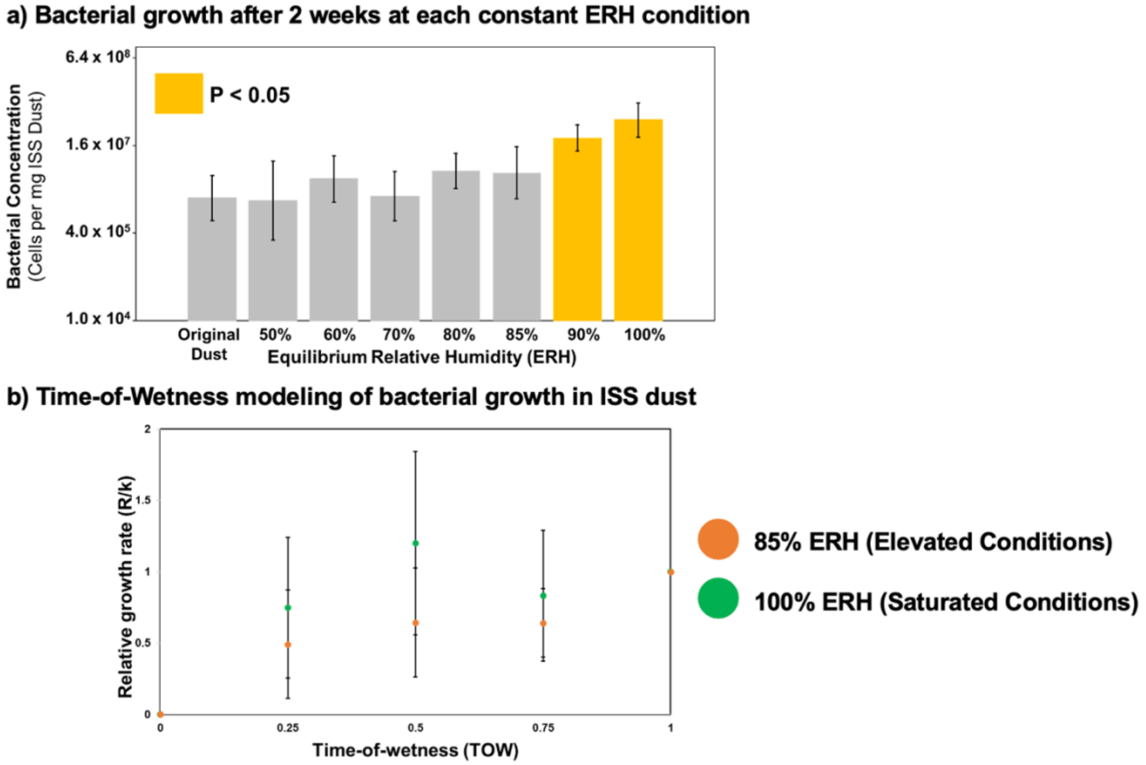

Supplemental Figure S1: A) Bacterial concentration of original dust and at each ERH condition tested (50, 60, 70, 80, 85, 90, and 100%) after two weeks at 25°C. Quantities for each condition represent a total of 36 qPCR measurements from 4 vacuum bags with triplicate physical samples from each bag and triplicate qPCR measurements per sample. B) Time-of-Wetness models for bacterial growth in ISS dust for elevated (85% ERH) and saturated (100% ERH) conditions.

Supplemental Table S2: Fungal and bacterial concentrations for 2-week incubation samples at 25°C and each ERH condition tested.

Equilibrium Relative Humidity Condition	Vacuum Bag	Fungal Concentration (SE/mg dust)	Bacterial Concentration (cells/mg dust)
Original Dust	1	6.14E+04	2.96E+05
		4.69E+04	7.28E+05
		7.51E+04	7.03E+05
	2	2.17E+04	1.36E+06
		2.06E+05	1.11E+06
		4.93E+05	1.57E+06

	3	7.02E+06	3.26E+05
		2.59E+06	4.73E+05
		1.51E+06	9.87E+04
	4	3.09E+07	7.85E+07
		3.50E+06	2.86E+07
		6.30E+06	1.32E+07
50%	1	1.66E+06	1.94E+06
		1.43E+06	1.83E+07
		2.38E+06	3.66E+07
	2	2.04E+04	2.73E+06
		1.33E+04	2.22E+06
		1.75E+04	2.19E+06
	3	7.84E+05	1.83E+03
		2.12E+06	1.07E+03
		4.43E+06	1.51E+04
	4	1.49E+07	3.57E+07
		5.42E+06	1.03E+07
		1.75E+07	1.97E+07
60%	1	1.15E+06	4.57E+06
		1.57E+06	3.26E+06
		2.44E+06	1.16E+06
	2	2.60E+04	7.25E+06
		1.33E+03	8.17E+04
		7.32E+04	3.95E+07
	3	2.62E+06	9.95E+03
		2.34E+06	3.33E+04
		1.18E+07	5.13E+05
	4	7.06E+06	1.48E+07
		9.47E+06	3.30E+07
		1.29E+07	3.24E+07
70%	1	1.33E+06	3.92E+06

		1.50E+06	1.35E+06
		6.26E+05	5.81E+05
	2	3.43E+04	3.59E+06
		6.37E+04	7.31E+06
		4.09E+03	4.19E+06
	3	2.92E+06	9.19E+03
		1.65E+06	5.69E+04
		3.00E+06	1.38E+05
	4	5.23E+06	6.18E+06
		9.50E+06	3.51E+07
		4.12E+06	1.56E+07
80%	1	9.64E+07	2.39E+06
		8.69E+06	3.14E+06
		7.58E+07	1.82E+07
	2	2.51E+04	4.71E+05
		1.14E+04	8.43E+05
		1.30E+04	2.07E+05
	3	1.83E+08	3.38E+03
		4.45E+07	2.09E+07
		5.06E+07	4.45E+04
	4	1.64E+07	1.37E+07
		7.75E+07	1.95E+07
		7.75E+07	6.10E+07
85%	1	1.81E+08	7.61E+05
		5.71E+08	1.56E+06
		2.38E+08	1.48E+06
	2	1.18E+06	2.54E+05
		6.83E+06	1.38E+06
		5.61E+06	2.25E+05
	3	3.82E+09	4.82E+06
		5.02E+09	7.94E+06
		5.09E+09	1.94E+06

	4	1.04E+10	1.72E+08
		1.25E+10	2.39E+08
		9.60E+09	1.20E+08
90%	1	1.53E+10	3.08E+06
		1.67E+10	7.46E+06
		2.50E+10	3.20E+07
	2	3.17E+08	5.43E+07
		2.35E+08	5.81E+06
		3.29E+08	6.09E+06
	3	1.28E+10	1.32E+06
		1.50E+10	1.56E+07
		1.48E+10	8.78E+05
	4	1.04E+10	2.03E+08
		5.14E+09	1.13E+08
		6.10E+09	4.79E+07
100%	1	2.80E+10	9.91E+06
		2.31E+10	1.89E+07
		4.25E+10	5.31E+06
	2	6.19E+08	3.37E+07
		7.04E+08	3.75E+07
		4.85E+08	4.22E+07
	3	3.26E+10	2.83E+07
		3.91E+10	2.80E+07
		2.78E+10	1.07E+07
	4	2.11E+10	1.17E+09
		1.92E+10	6.78E+07
		1.64E+10	2.37E+08

Supplemental Table S3: Summary of Satterthwaite two-sample t-test statistics for fungal and bacterial 2-week incubations.

Satterthwaite Two-Sample ttest		
Original Dust vs:	Fungal P-value	Bacteria P-value
50% RH	0.3893	0.5996
60% RH	0.368	0.4377
70% RH	0.4554	0.5243
80% RH	0.0439	0.4594
85% RH	< 0.0001	0.1293
90% RH	< 0.0001	0.005
100% RH	< 0.0001	0.0001

Supplemental Table S4: qPCR values for fungal and bacterial quantities for frozen dust sample and the original dust collected from the ISS vacuum bags.

Sample	Fungal Quantity (spore equivalents per mg dust)	Bacterial Quantity (cells per mg dust)
Bag 1 Original Dust (Average)	6.12E+04	1.40E+05
Bag 2 Original Dust (Average)	2.17E+04	5.93E+05
Bag 3 Original Dust (Average)	1.24E+04	7.67E+04
Bag 4 Original Dust (Average)	1.88E+04	7.61E+06
Frozen NOD3 D3-01 GMT111	1.60E+03	2.74E+06
Frozen NOD3 D2-15 INLET GMT118	5.63E+02	9.90E+05
Frozen NOD3 D3-01 HEPA GRATE INLET GMT118	6.47E+02	7.39E+05
Frozen NOD1 D3-01 GMT111	2.60E+02	9.21E+05

Frozen NOD1 D3-01 HEPA GRATE INLET GMT118	1.77E+02	1.80E+05
Frozen LAB 15-D5 GMT111	7.68E+02	4.99E+05
Frozen LAB 101 IMV INLET GMT118	5.22E+02	1.69E+05
Frozen LAB S5D HEPA GMT118	1.40E+02	3.12E+05

Supplemental Table S5: Total fungal growth rates for TOW incubations. Values represent the average of the 4 ISS bags collected.

	Total Fungal Growth Rate (spore eq/mg dust/day)			
**TOW Condition**	**Day 5 - Day 10**	**Day 10 - Day 14**	**Day 14 -Day 21**	**Day 5 - Day 21**
50% RH, 24 hours	6.69E+03	0	4.96E+03	1.19E+03
85% RH, 6 hours	1.88E+04	0	2.53E+03	3.25E+03
85% RH, 12 hours	1.30E+04	0	2.54E+04	1.26E+04
85% RH, 18 hours	5.18E+04	9.77E+03	7.85E+04	5.30E+04
85% RH, 24 hours	2.83E+06	1.14E+07	9.52E+06	7.91E+06
100% RH, 6 hours	0	7.65E+04	1.39E+05	6.67E+04
100% RH, 12 hours	7.27E+04	1.50E+06	1.90E+06	1.23E+06
100% RH, 18 hours	1.42E+07	4.02E+07	2.77E+07	2.66E+07
100% RH, 24 hours	4.22E+07	1.14E+07	1.38E+07	2.21E+07

Supplemental Table S6: qPCR values for all TOW samples

**Condition**	**Bag**	**TOW**	**Day**	**Fungal Concentration (Spore equivalents/mg Dust)**	**Bacterial Concentration (Cells/mg Dust)**
Elevated (85% ERH)	1	6	5	5.05E+05	1.97E+06
			10	8.75E+05	8.31E+05
			14	6.29E+05	1.01E+06
			21	6.44E+05	6.66E+05
		12	5	5.99E+05	1.22E+07
			10	1.22E+06	2.80E+06

			14	1.13E+05	1.60E+06
			21	1.88E+05	1.90E+06
		18	5	1.01E+05	5.72E+05
			10	8.99E+05	1.03E+06
			14	9.39E+05	1.67E+06
			21	5.61E+05	6.54E+05
		24	5	4.35E+04	3.82E+06
			10	3.78E+06	1.79E+06
			14	8.43E+06	9.42E+05
			21	1.50E+08	4.53E+06
	2	6	5	1.19E+05	5.21E+05
			10	1.19E+05	3.97E+05
			14	1.28E+05	2.58E+05
			21	1.92E+05	1.57E+05
		12	5	5.49E+05	2.26E+06
			10	1.84E+05	1.82E+06
			14	1.13E+06	8.33E+06
			21	1.74E+06	2.22E+06
		18	5	1.15E+05	7.72E+05
			10	2.95E+05	4.41E+06
			14	3.85E+05	1.37E+06
			21	2.15E+06	8.26E+05
		24	5	2.77E+05	1.33E+07
			10	4.80E+07	3.11E+07
			14	2.23E+08	3.37E+07
			21	3.38E+08	1.80E+07
	3	6	5	7.23E+03	2.89E+07
			10	1.54E+04	5.22E+07
			14	6.24E+03	7.56E+07
			21	5.70E+03	4.12E+07
		12	5	3.82E+02	1.67E+07

			10	1.04E+03	9.25E+07
			14	7.48E+02	4.26E+07
			21	2.04E+04	5.92E+07
		18	5	1.82E+03	3.49E+07
			10	2.71E+04	1.37E+07
			14	3.61E+04	1.71E+07
			21	4.22E+05	1.44E+07
		24	5	4.03E+03	5.09E+07
			10	1.65E+06	1.10E+08
			14	3.38E+06	6.10E+07
			21	9.95E+06	1.32E+08
	4	6	5	5.87E+03	3.42E+07
			10	3.35E+03	3.29E+07
			14	1.05E+04	1.77E+07
			21	3.66E+03	1.78E+07
		12	5	3.14E+03	4.40E+08
			10	3.08E+03	4.48E+07
			14	4.34E+03	5.50E+07
			21	4.65E+03	6.31E+07
		18	5	8.47E+02	1.84E+07
			10	3.37E+04	6.66E+07
			14	5.20E+04	3.55E+07
			21	4.83E+05	5.15E+08
		24	5	1.05E+04	1.02E+08
			10	3.49E+06	9.17E+07
			14	4.66E+06	7.72E+07
			21	7.61E+06	6.89E+07
Saturated (100% ERH)	1	6	5	1.13E+06	1.23E+06
			10	2.63E+05	1.05E+06
			14	9.69E+05	7.13E+05
			21	3.55E+06	7.07E+05

		12	5	3.22E+05	1.17E+06
			10	7.33E+04	8.26E+05
			14	2.95E+06	6.81E+05
			21	1.30E+07	3.02E+05
		18	5	2.28E+07	1.00E+06
			10	2.30E+08	6.14E+05
			14	7.91E+08	2.30E+06
			21	1.37E+09	9.02E+05
		24	5	2.16E+08	7.38E+06
			10	5.44E+08	1.72E+07
			14	6.80E+08	1.88E+07
			21	6.35E+08	4.53E+07
	2	6	5	2.16E+04	4.63E+05
			10	2.04E+04	1.56E+05
			14	5.58E+05	1.71E+06
			21	1.63E+06	1.41E+06
		12	5	4.20E+05	8.83E+05
			10	1.33E+06	1.41E+06
			14	2.06E+07	5.62E+05
			21	5.92E+07	7.87E+05
		18	5	1.09E+08	8.05E+06
			10	1.76E+08	1.81E+07
			14	2.54E+08	1.20E+06
			21	4.34E+08	7.87E+05
		24	5	9.48E+07	2.93E+06
			10	6.01E+08	7.88E+06
			14	6.48E+08	1.49E+07
			21	1.07E+09	4.31E+07
	3	6	5	7.53E+02	1.40E+07
			10	1.31E+04	3.59E+07
			14	1.55E+04	6.03E+07

			21	9.11E+04	2.55E+07
		12	5	1.34E+04	2.71E+06
			10	3.64E+05	2.33E+06
			14	1.65E+06	4.85E+06
			21	4.18E+06	4.58E+06
		18	5	2.14E+06	2.65E+07
			10	6.85E+06	4.75E+07
			14	9.51E+06	1.76E+07
			21	2.07E+07	5.92E+07
		24	5	9.99E+06	1.36E+08
			10	1.93E+07	1.62E+08
			14	2.23E+07	9.70E+07
			21	1.53E+07	6.62E+07
	4	6	5	1.00E+03	1.59E+07
			10	2.33E+04	3.79E+07
			14	2.00E+03	7.75E+06
			21	1.54E+05	5.69E+07
		12	5	4.02E+04	2.14E+07
			10	4.78E+05	1.63E+07
			14	1.01E+06	3.34E+07
			21	3.00E+06	3.94E+08
		18	5	9.90E+05	1.46E+07
			10	5.26E+06	3.61E+07
			14	6.35E+06	4.49E+07
			21	8.99E+06	4.09E+07
		24	5	6.33E+06	5.67E+07
			10	1.16E+07	8.44E+07
			14	1.13E+07	5.56E+08
			21	1.36E+07	8.91E+07
Unmodified (50% ERH)	1	24	5	3.35E+04	6.96E+07
			10	2.39E+04	1.17E+08
			14	8.98E+03	8.89E+07

			21	3.86E+04	1.09E+08
	2	24	5	8.82E+04	1.03E+04
			10	1.78E+05	9.88E+03
			14	7.24E+04	8.85E+04
			21	1.71E+05	9.00E+03
	3	24	5	8.95E+02	7.45E+03
			10	2.28E+03	1.65E+04
			14	1.04E+03	2.12E+04
			21	9.93E+01	1.88E+04
	4	24	5	7.25E+03	3.14E+04
			10	2.16E+03	7.72E+03
			14	2.25E+03	1.37E+04
			21	2.14E+03	1.06E+04

Supplemental Table S7: Effective growth rate constants (k) for TOW at constant (24 hours per day) ERH conditions

Condition	Bag	Fungal Growth Rate Constant (k) day^{-1}	Bacterial Growth Rate (k) day^{-1}
Elevated (85% ERH)	1	2.0883	0.2729
	2	2.6005	0.6109
	3	2.3404	1.2368
	4	2.2270	0.0808
Saturated (100% ERH)	1	1.9641	0.4745
	2	2.3545	0.2664
	3	1.2885	0.5486
	4	1.0897	0.2811
Unmodified (50% ERH)	1	0.1363	0.1080
	2	0.3937	0.1790
	3	0	0.3038
	4	0	0

Supplemental Table S8: Effective growth constants (R) for all TOW samples.

Condition	Bag	TOW	Fungal Effective Growth Constant (k) day^{-1}	Bacterial Effective Growth Constant (k) day^{-1}
Elevated (85% ERH)	1	6	0.4927	0
		12	0.0580	0.0357
		18	0.6663	0.1331
	2	6	0.4436	0
		12	1.2930	0.2295
		18	1.0405	0
	3	6	0.1579	1.0814
		12	0.4963	1.1516
		18	1.3337	0.7030
	4	6	0.1469	0
		12	0.169	0
		18	1.4775	0.5764
Saturated (100% ERH)	1	6	0.7963	0
		12	0.9499	0
		18	2.3585	0.1730
	2	6	1.1897	0.1395
		12	1.9720	0.0000
		18	2.0660	0.0000
	3	6	1.0310	1.0354
		12	1.9749	1.3814
		18	1.9627	1.0164
	4	6	0.9060	0.0000
		12	1.7531	0.5017
		18	1.8361	0.1168

Supplemental Table S9: Relative growth constants (R/k) for all TOW samples.

Condition	Bag	TOW	Fungal Relative Growth Constant (R/k)	Bacterial Relative Growth Constant (R/k)
Elevated (85% ERH)	1	6	0.2359	0
		12	0.0278	0.0649
		18	0.3191	0.2418
	2	6	0.1706	0
		12	0.4972	0.4170
		18	0.4001	0
	3	6	0.0675	1.9649
		12	0.2121	2.0925
		18	0.5699	1.2774
	4	6	0.0660	0
		12	0.0759	0
		18	0.6634	1.0473
Saturated (100% ERH)	1	6	0.4054	0
		12	0.4836	0
		18	1.2008	0.4406
	2	6	0.5053	0
		12	0.8375	0.3553
		18	0.8775	0
	3	6	0.8002	2.6369
		12	1.5327	3.5182
		18	1.5232	2.5886
	4	6	0.8314	0
		12	1.6088	1.2778
		18	1.6850	0.2975

Supplemental Table S10: Most common taxa that was present in all sequenced samples sorted by order, genus, and species for bacteria and fungi.

Name	Present in % of Samples	Name	Present in % of Samples
**Fungal Order**		**Bacterial Order**	
Eurotiales	100	*Bacillales*	99
Helotiales	95	*Actinomycetales*	99
Sporidiobolales	94	*Clostridiales*	96
Saccharomycetales	88	*Lactobacillales*	92
Pleosporales	78	*Pseudomonadales*	84
Hypocreales	75	*Gemellales*	80
Tremellales	71	*Pasteurellales*	75
Malasseziales	64	*Fusobacteriales*	75
Incertae sedis	61	*Streptophyta*	74
Capnodiales	61	*Bacteroidales*	74
**Fungal Genus**		**Bacterial Genus**	
Aspergillus	100	*Staphylococcus*	99
Penicillium	95	*Corynebacterium*	99
Rhodotorula	94	*Anaerococcus*	87
Candida	79	*Streptococcus*	85
Cyberlindnera	79	*Finegoldia*	82
Fusarium	70	*Brevibacterium*	79
Alternaria	69	*Actinomyces*	78
Gibberella	66	*Pseudomonas*	78
Malassezia	64	*Lactobacillus*	77
Talaromyces	61	*Micrococcus*	76
**Fungal Species**		**Bacterial Species**	

Aspergillus sydowii	100	*Corynebacterium kroppenstedtii*	81
Aspergillus unguis	100	*Staphylococcus pettenkoferi*	78
Penicillium chrysogenum	90	*Lactobacillus helveticus*	74
Aspergillus hongkongensis	85	*Acinetobacter rhizosphaerae*	70
Aspergillus nidulans	84	*Veillonella parvula*	70
Cyberlindnera jadinii	81	*Veillonella dispar*	69
Aspergillus ruber	77	*Haemophilus parainfluenzae*	67
Candida tropicalis	72	*Corynebacterium stationis*	65
Penicillium corylophilum	70	*Faecalibacterium prausnitzii*	65
Rhodotorula dairenensis	69	*Rothia dentocariosa*	61
Rhodotorula mucilaginosa	69	*Corynebacterium variabile*	60
Penicillium gladioli	67	*Streptococcus infantis*	60
Fusarium acutatum	66	*Staphylococcus saprophyticus*	60
Gibberella intricans	66	*Corynebacterium durum*	60
Talaromyces minioluteus	61	*Streptococcus anginosus*	60
Aspergillus penicillioides	61	*Capnocytophaga ochracea*	58
Malassezia restricta	60	*Prevotella melaninogenica*	57
Alternaria alternata	60	*Aggregatibacter segnis*	53

Candida albicans	55	*Sphingobium yanoikuyae*	52
Malassezia globosa	54	*Campylobacter ureolyticus*	51

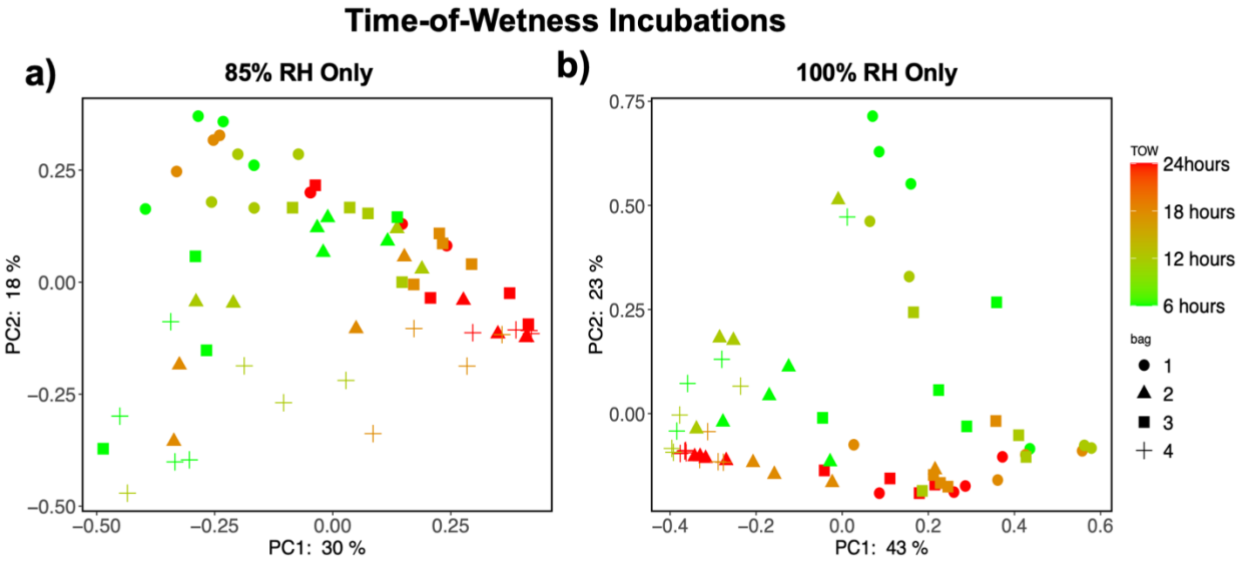

Supplemental Figure S2: Fungal principal coordinate analyses of time-of-wetness samples separated out by elevated (A) and saturated (B) relative humidity conditions.

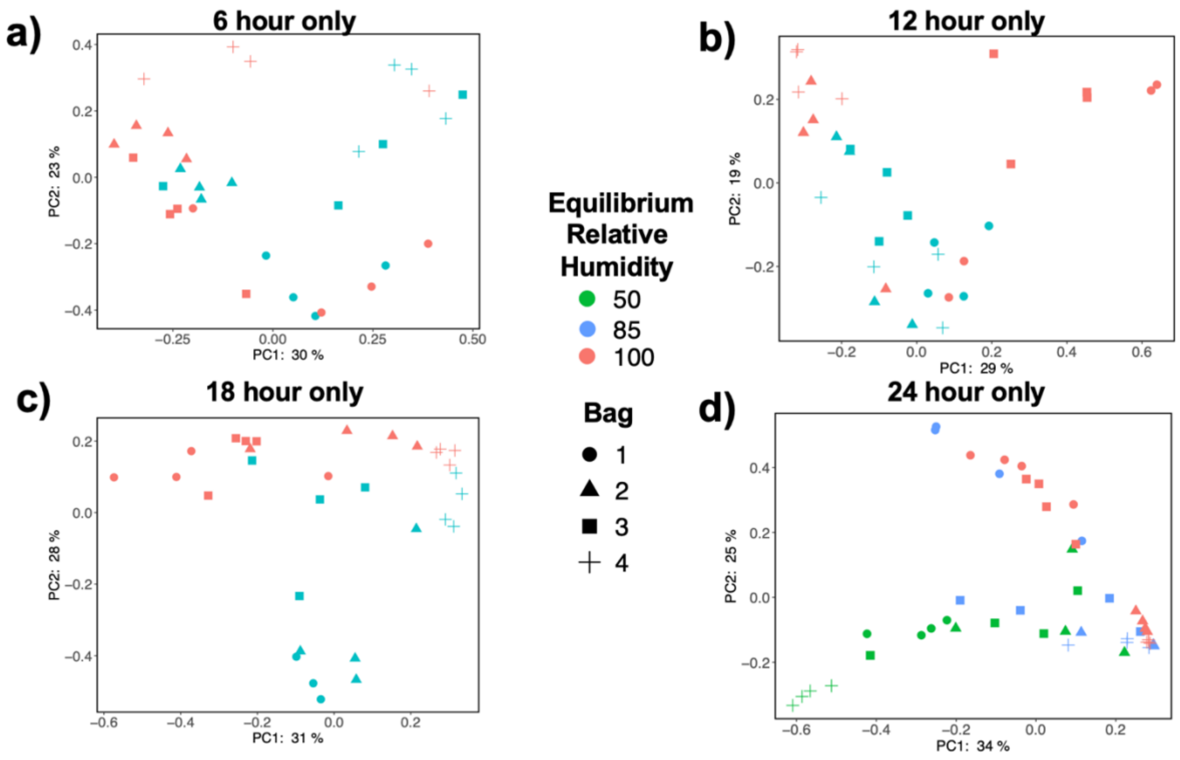

Supplemental Figure S3: Fungal principal coordinate analyses of time-of-wetness samples separated out by time points of 6 hours (A), 12 hours (B), 18 hours (C), and 24 hours (D).

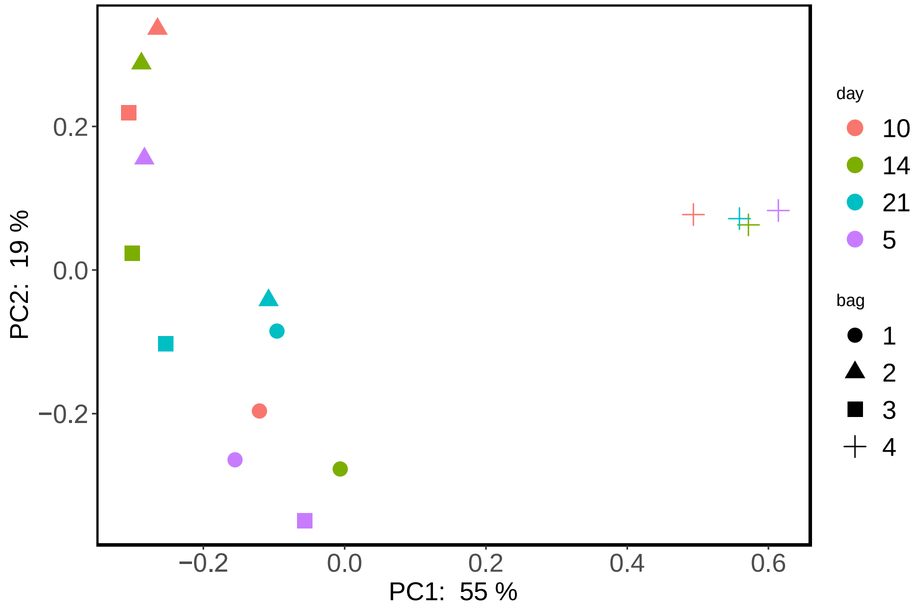

Supplemental Figure S4: Fungal principal coordinate analyses of 50% ERH time-of-wetness samples separated out by sample days.

Supplemental Table S11: Adonis values for fungal bray Curtis PCoA analysis for each time-of-wetness condition.

Variable	R^2	P-value
**50% RH only**		
Bag	0.288	0.010
Day	0.013	0.927
**85% RH only**		
Bag	0.121	0.001
Time of Wetness	0.159	0.001
Day	0.007	0.793
**100% RH only**		
Bag	0.145	0.001
Time of Wetness	0.090	0.002
Day	0.025	0.086
**6-hour TOW only**		
Bag	0.163	0.001
Relative Humidity	0.072	0.019
Day	0.036	0.198
**12-hour TOW only**		
Bag	0.123	0.001
Relative Humidity	0.111	0.001
Day	0.018	0.702
**18-hour TOW only**		
Bag	0.175	0.001
Relative Humidity	0.185	0.001
Day	0.009	0.838
**24-hour TOW only**		
Bag	0.104	0.001

Relative Humidity	0.201	0.001
Day	0.008	0.786

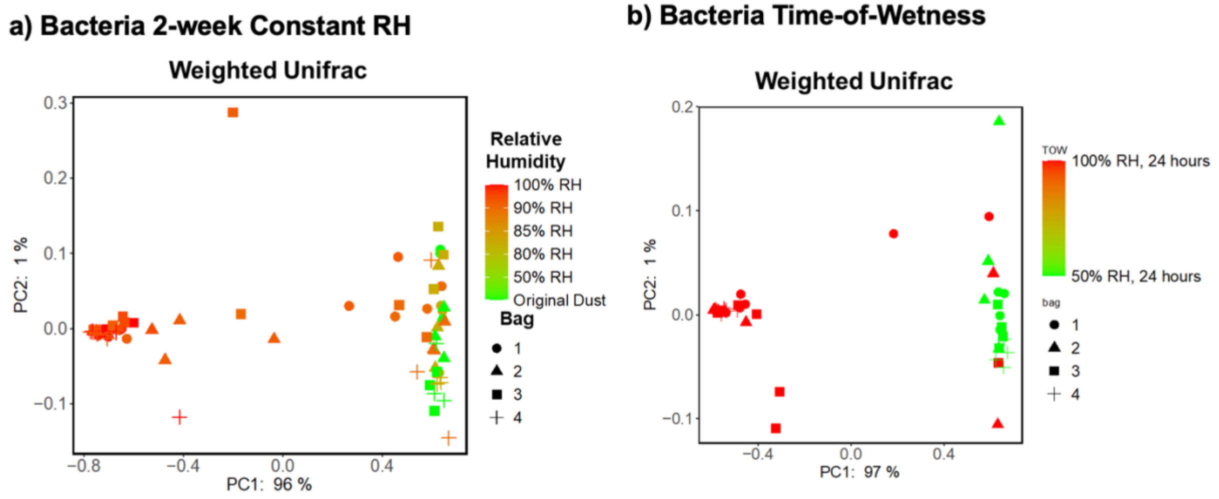

Supplemental Figure S5: A) Bacterial PCoA plots for constant ERH 2-week incubations. Only original dust, 50%, 80%, 85%, 90%, and 100% ERH samples were sequenced. B) Bacterial PCoA plot for Time-of-Wetness incubations. Only constant 24-hour samples for 50% and 100% ERH conditions were performed for days 5, 10, 14, and 21. Both figures represent weighted unifrac distance matrices.

Supplemental Table 12: Adonis statistics for bacterial time of wetness beta diversity measurements.

**Variable**	**R^2**	**P-value**
**Bacterial Time of Wetness (50% and 100% RH for 24 hours only)**		
Bag	0.050	0.008
RH	0.282	0.001
Day	0.039	0.016
**Bacterial 2-week Incubations (Original Dust, 50%, 80%, 85%, 90%, and 100% RH)**		
Bag	0.038	0.018
RH	0.218	0.001

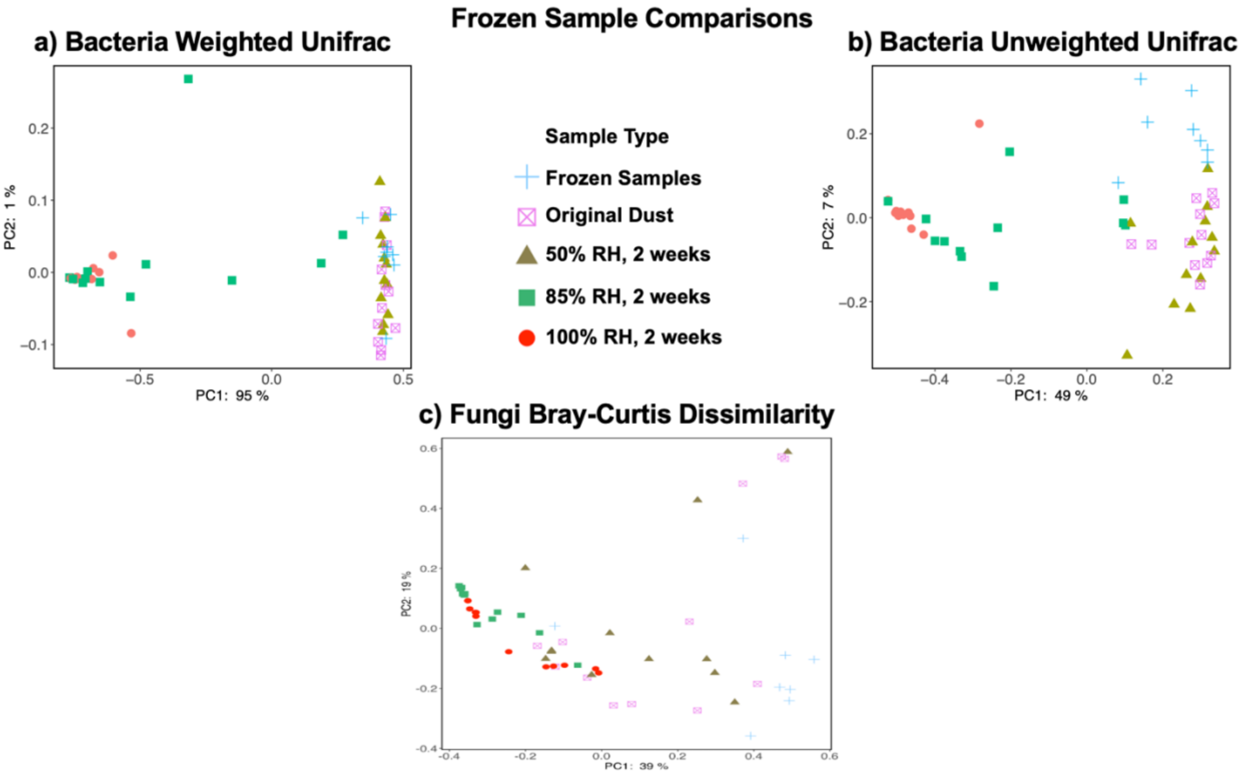

Supplemental Figure S6: Principal coordinate analyses of frozen dust sample returned from the ISS. Frozen samples were compared to original dust samples (from ISS vacuum bag) as well as 2-week incubations at 50%, 85%, and 100% ERH. Fungi PCoA plots used the Bray-Curtis dissimilarity statistics (C), while bacteria used both weighted (A) and unweighted unifrac (B).

Supplemental Table S13: Adonis statistics for fungal and bacterial frozen sample comparisons. Frozen samples were compared to original dust samples (from ISS vacuum bag) as well as 2-week incubations at 50%, 85%, and 100% RH.

**Frozen Sample Comparison Adonis**		
Comparison	R^2	P-value
Fungal Bray-Curtis Dissimilarity	0.361	0.001
Bacterial Unweighted Unifrac	0.505	0.001
Bacterial Weighted Unifrac	0.876	0.001

Supplemental Table S14: Fungal alpha diversity Kruskal-Wallis statistics for richness and Shannon diversity for 2-week incubations at each RH conditions tested. Significant changes in both richness and Shannon diversity compared to the original dust began to occur at 80% RH (Q<0.05).

**Condition**	**Richness**			**Shannon Diversity**		
**Original Dust vs.**	**H**	**P-value**	**Q-value**	**H**	**P-value**	**Q-value**
50% RH	1.33	0.2481	0.3158	0.85	0.3556	0.4526
60% RH	0.80	0.3706	0.4324	0.27	0.6033	0.6757
70% RH	1.14	0.2852	0.3472	1.08	0.2987	0.3983
80% RH	1.69	0.1938	0.2584	5.07	0.0243	0.0426
85% RH	6.91	0.0086	0.0134	9.01	0.0027	0.0075
90% RH	10.83	0.0001	0.0023	8.33	0.0039	0.0091
100% RH	4.33	0.0374	0.0551	7.68	0.0056	0.0120

Supplemental Table S15: Richness and Shannon diversity Kruskal-Wallis statistics for fungal time-of-wetness samples.

	**Richness**			**Shannon Diversity**		
**Variable**	**H**	**P-value**	**Q-value**	**H**	**P-value**	**Q-value**
**Fungal Time-of-Wetness 50% ERH, 24 hours only**						
Day 5 vs. Day 10	0.83	0.7728	0.9274	0.33	0.5637	1
Day 5 vs. Day 14	0.75	0.3865	0.9274	0.83	0.7728	1
Day 5 vs. Day 21	0.75	0.3865	0.9274	0	1	1

**Fungal Time-of-Wetness 85% ERH Only**						
6 hours vs. 12 hours	1.84	0.1748	0.2098	0.46	0.4975	0.4975
6 hours vs. 18 hours	2.14	0.1436	0.2098	2.76	0.0969	0.1261
6 hours vs. 24 hours	17.52	< 0.0001	< 0.0001	18.79	< 0.0001	< 0.0001
**Fungal Time-of-Wetness 100% ERH Only**						
6 hours vs. 12 hours	7.78	0.0053	0.0106	6.76	0.0093	0.0186
6 hours vs. 18 hours	11.66	0.0001	0.0038	7.57	0.0059	0.0178
6 hours vs. 24 hours	9.22	0.0024	0.0072	8.20	0.0042	0.0178
**Fungal Time-of-Wetness 6 hour Only**						
85% vs. 100% ERH	6.00	0.0143	0.0143	6.19	0.0129	0.129
**Fungal Time-of-Wetness 12 hour Only**						
85% vs. 100% ERH	12.57	< 0.0001	< 0.0001	12.02	0.0001	0.0001
**Fungal Time-of-Wetness 18 hour Only**						
85% vs. 100% ERH	21.22	< 0.0001	< 0.0001	9.51	0.0020	0.0020
**Fungal Time-of-Wetness 24 hour Only**						
50% vs 85% ERH	17.20	< 0.0001	< 0.0001	11.25	0.0010	0.0012
50% vs 100% ERH	20.14	< 0.0001	< 0.0001	13.64	0.0002	0.0007

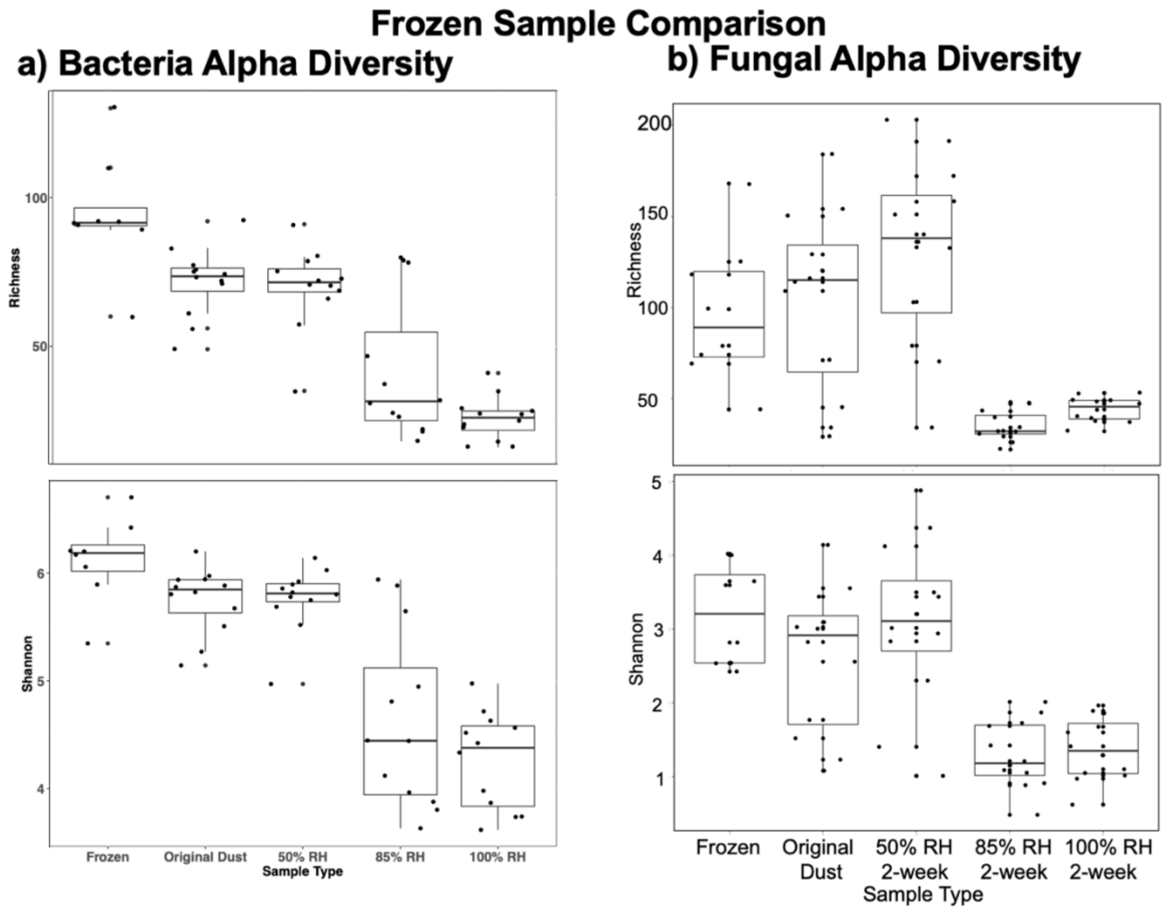

Supplemental Figure S7: Frozen sample alpha diversity plots for (A) bacteria and (B) fungi. Frozen dust samples were compared to original dust, 50% ERH 2-week, 85% ERH 2-week, and 100% ERH 2-week incubations.

Supplemental Table S16: Kruskal-Wallis test statistics for alpha diversity metrics for all sequenced bacterial samples.

	**Richness**			**Shannon Diversity**		
Condition	H	P-value	Q-value	H	P-value	Q-value
**Bacteria 2-week Incubations**						
OD vs. 50% RH	0.08	0.7726	0.8278	0.08	0.7728	0.7728

OD vs. 80% RH	0.08	0.7724	0.8278	0.12	0.7290	0.7728
OD vs. 85% RH	8.72	0.0031	0.0052	10.83	0.0001	0.0021
OD vs. 90% RH	17.30	< 0.0001	0.0001	17.28	< 0.0001	0.0002
OD vs. 100% RH	17.30	< 0.0001	0.0001	17.28	< 0.0001	0.0002
**Bacteria Time-of-Wetness Incubations**						
50% vs 100% RH, 24 hours	22.56	< 0.0001	< 0.0001	22.11	< 0.0001	< 0.0001

Supplemental Table S17: Kruskal-Wallis test statistics for fungal and bacterial frozen sample comparisons.

	**Richness**			**Shannon Diversity**		
**Fungi**						
Frozen vs.	H	P-value	Q-value	H	P-value	Q-value
Original Dust	0.15	0.6997	0.6697	1.01	0.3159	0.4445
50% RH, 2-week	2.75	0.0970	0.1213	0	1	1
85% RH, 2-week	12.63	0.0004	0.0014	13.71	0.0002	0.0011
100% RH, 2-week	10.27	0.0013	0.0027	13.71	0.0002	0.0011
**Bacteria**						

Frozen vs.	H	P-value	Q-value	H	P-value	Q-value
Original Dust	6.91	0.0086	0.0143	6.10	0.0136	0.0169
50% RH, 2-week	7.74	0.0054	0.0108	6.10	0.0136	0.0169
85% RH, 2-week	12.07	0.0005	0.0013	11.52	0.0001	0.0017
100% RH, 2-week	13.76	0.0002	0.0007	13.71	0.0002	0.0001

Supplemental Table S18: Differential abundance fungal comparison between non-elevated (Original Dust, 50, 60, 70% RH) and elevated (80, 85, 90, 100% RH) after 2-week constant ERH incubations at 25°C

Fungal Species	Unadjusted Pvalue	Adjusted FDR P-value
**More abundant at non-elevated RH conditions**		
Candida tropicalis	<.0001	<.0001
Rhodotorula dairenensis	<.0001	<.0001
Rhodotorula mucilaginosa	<.0001	<.0001
Fusarium acutatum	<.0001	<.0001
Gibberella intricans	<.0001	<.0001
Talaromyces minioluteus	<.0001	<.0001
Malassezia restricta	<.0001	<.0001
Alternaria alternata	<.0001	<.0001
Candida albicans	<.0001	<.0001
Malassezia globosa	<.0001	<.0001
Cladosporium delicatulum	<.0001	<.0001
Verticillium dahliae	<.0001	<.0001
Papiliotrema laurentii	<.0001	<.0001
Fusarium culmorum	<.0001	<.0001
Candida parapsilosis	<.0001	<.0001
Debaryomyces hansenii	<.0001	<.0001
Aspergillus conicus	<.0001	<.0001
Epicoccum nigrum	<.0001	<.0001
Mycosphaerella tassiana	<.0001	<.0001

Candida hyderabadensis	<.0001	<.0001
Malassezia sympodialis	<.0001	<.0001
Naganishia diffluens	<.0001	<.0001
Alternaria brassicae	<.0001	<.0001
Nigrospora oryzae	<.0001	<.0001
Rhodosporidiobolus fluvialis	<.0001	<.0001
Wallemia tropicalis	<.0001	<.0001
Colletotrichum gloeosporioides	<.0001	<.0001
Naganishia albida	<.0001	<.0001
Aureobasidium namibiae	<.0001	<.0001
Alternaria nepalensis	<.0001	<.0001
Malassezia caprae	<.0001	<.0001
Vishniacozyma victoriae	<.0001	<.0001
Alternaria metachromatica	<.0001	<.0001
Cyberlindnera jadinii	0.001	0.0019
Malassezia dermatis	<.0001	0.0001
Malassezia pachydermatis	<.0001	0.0001
Candida sake	<.0001	0.0002
Alternaria terricola	<.0001	0.0002
Zygosaccharomyces rouxii	0.0001	0.0003
Penicillium glabrum	0.0002	0.0004
Agaricus bisporus	0.0002	0.0005
Acremonium charticola	0.0002	0.0005
Malassezia arunalokei	0.0003	0.0006
Trichosporon asahii	0.0003	0.0006
Septoria cretae	0.0003	0.0006
Cladosporium halotolerans	0.0004	0.0008
Phaeosphaeria podocarpi	0.0005	0.0009
Malassezia cuniculi	0.0006	0.0012
Dekkera custersiana	0.0006	0.0012
Aspergillus restrictus	0.0006	0.0012
Lasiodiplodia brasiliensis	0.0007	0.0014
Colletotrichum truncatum	0.0008	0.0015
Saccharomyces cerevisiae	0.0013	0.0023
Corynespora torulosa	0.0021	0.0036
Schizophyllum amplum	0.0021	0.0036
Filobasidium magnum	0.0026	0.0042

Bipolaris sorokiniana	0.0025	0.0042
Symmetrospora vermiculata	0.0025	0.0042
Stagonosporopsis dorenboschii	0.0027	0.0043
Pyrenochaetopsis leptospora	0.0028	0.0045
Penicillium sclerotigenum	0.0043	0.0067
Xenodidymella humicola	0.0043	0.0067
Penicillium expansum	0.005	0.0076
Leptospora rubella	0.0055	0.0082
Penicillium decumbens	0.0056	0.0083
Vishniacozyma heimaeyensis	0.0061	0.0088
Plectosphaerella oratosquillae	0.0069	0.0099
Sarocladium implicatum	0.0071	0.0101
Aspergillus flavus	0.0094	0.0131
Cystobasidium slooffiae	0.0137	0.0189
Cystobasidium pinicola	0.0159	0.0213
Trichothecium roseum	0.0158	0.0213
Alternaria didymospora	0.02	0.0265
Penicillium brevicompactum	0.0211	0.0273
Paramyrothecium roridum	0.0212	0.0273
Cladosporium sphaerospermum	0.0287	0.0367
Penicillium thymicola	0.0304	0.0384
**More abundant at elevated RH conditions**		
Aspergillus sydowii	<.0001	<.0001
Aspergillus unguis	<.0001	<.0001
Aspergillus nidulans	<.0001	<.0001
Aspergillus subversicolor	<.0001	<.0001
Penicillium chrysogenum	0.0009	0.0016
Aspergillus hongkongensis	0.0046	0.0071

Supplemental Table S19: Differential abundance fungal comparison between original dust samples and 24-hour TOW saturated (100% RH) conditions. There were 52 fungal species more abundant in the original dust compared to 6 species more abundant at saturated conditions.

Fungal Species	Unadjusted P-value	Adjusted FDR P-value
**More abundant in Original Dust (52 species)**		
Gibberella intricans	<.0001	<.0001
Malassezia restricta	<.0001	<.0001

Malassezia globosa	<.0001	<.0001
Cladosporium delicatulum	<.0001	<.0001
Papiliotrema laurentii	<.0001	<.0001
Fusarium culmorum	<.0001	<.0001
Cladosporium halotolerans	<.0001	<.0001
Cyberlindnera jadinii	<.0001	<.0001
Alternaria alternata	<.0001	0.0001
Fusarium acutatum	<.0001	0.0002
Talaromyces minioluteus	<.0001	0.0002
Aspergillus penicillioides	<.0001	0.0002
Epicoccum nigrum	<.0001	0.0002
Malassezia sympodialis	<.0001	0.0002
Naganishia diffluens	<.0001	0.0002
Aspergillus conicus	<.0001	0.0004
Mycosphaerella tassiana	<.0001	0.0004
Candida albicans	0.0001	0.0005
Wallemia tropicalis	0.0001	0.0006
Candida parapsilosis	0.0008	0.0031
Rhodotorula mucilaginosa	0.0008	0.0032
Nigrospora oryzae	0.0008	0.0032
Debaryomyces hansenii	0.0014	0.005
Stagonosporopsis dorenboschii	0.0017	0.0059
Rhodosporidiobolus fluvialis	0.0021	0.007
Penicillium glabrum	0.0021	0.007
Corynespora torulosa	0.0022	0.0071
Verticillium dahliae	0.0024	0.0075
Candida hyderabadensis	0.0025	0.0075
Aureobasidium namibiae	0.0027	0.008
Malassezia dermatis	0.003	0.0087
Vishniacozyma victoriae	0.0037	0.0104
Candida tropicalis	0.005	0.0132
Rhodotorula dairenensis	0.0049	0.0132
Alternaria brassicae	0.0057	0.0148
Penicillium sclerotigenum	0.0065	0.0161
Malassezia arunalokei	0.0069	0.0167
Malassezia caprae	0.0097	0.0229
Agaricus bisporus	0.0115	0.0264
Colletotrichum gloeosporioides	0.0132	0.0297
Dekkera custersiana	0.0134	0.0297
Lasiodiplodia brasiliensis	0.0149	0.0323

Phaeosphaeria podocarpi	0.0155	0.0328
Penicillium thymicola	0.0211	0.0438
Candida sake	0.0215	0.0438
**More abundant at 100% RH 24 hr TOW only (6 species)**		
Aspergillus sydowii	<.0001	<.0001
Aspergillus unguis	<.0001	<.0001
Penicillium chrysogenum	<.0001	<.0001
Aspergillus nidulans	<.0001	<.0001
Aspergillus subversicolor	<.0001	<.0001
Penicillium gladioli	<.0001	0.0002

Supplemental Table S20: Differential abundance fungal comparison between unmodified (50% RH) 24-hour TOW samples and 24-hour TOW saturated (100% RH) conditions. There were 29 fungal species more abundant in the unmodified condition compared to 10 species more abundant at saturated conditions.

**Fungal Species More abundant at unmodified (50% ERH) conditions (29 species)**	**Unadjusted P-value**	**Adjusted FDR P-value**
Cladosporium delicatulum	<.0001	0.0005
Malassezia restricta	<.0001	0.0007
Alternaria alternata	0.0001	0.0013
Epicoccum nigrum	0.0003	0.0032
Aspergillus penicillioides	0.0005	0.0042
Nigrospora oryzae	0.0006	0.0042
Malassezia globosa	0.0006	0.0044
Gibberella intricans	0.0011	0.0072
Wallemia tropicalis	0.0013	0.0079
Verticillium dahliae	0.0015	0.0086
Malassezia sympodialis	0.0016	0.009
Candida albicans	0.0022	0.011
Fusarium culmorum	0.0021	0.011
Mycosphaerella tassiana	0.0028	0.0135
Cyberlindnera jadinii	0.004	0.017
Papiliotrema laurentii	0.004	0.017
Penicillium sclerotigenum	0.0051	0.0208
Alternaria brassicae	0.0059	0.0224
Saccharomyces cerevisiae	0.0079	0.0288
Malassezia dermatis	0.0095	0.0325
Aspergillus conicus	0.0106	0.035
Fusarium acutatum	0.0116	0.0361
Alternaria nepalensis	0.0114	0.0361

Debaryomyces hansenii	0.012	0.0362
Malassezia arunalokei	0.0141	0.0415
Stagonosporopsis dorenboschii	0.0157	0.0432
Penicillium glabrum	0.0159	0.0432
Vishniacozyma victoriae	0.0152	0.0432
Aureobasidium namibiae	0.0171	0.0453
**More abundant at saturated (100% ERH) conditions (10 species)**		
Aspergillus sydowii	<.0001	<.0001
Aspergillus unguis	<.0001	<.0001
Penicillium chrysogenum	<.0001	<.0001
Aspergillus nidulans	<.0001	<.0001
Penicillium gladioli	<.0001	<.0001
Aspergillus subversicolor	<.0001	<.0001
Aspergillus hongkongensis	0.0004	0.0033
Aspergillus puniceus	0.0033	0.0153
Penicillium concentricum	0.0056	0.0219
Aspergillus flavus	0.0085	0.0299

Supplemental Table S21: Differential abundance fungal comparison between high (85% RH) 24-hour TOW samples and 24-hour TOW saturated (100% RH) conditions. There was 1 fungal species more abundant in the high condition compared to 8 species more abundant at saturated conditions.

Fungal Species	Unadjusted P-value	Adjusted FDR P-value
**More abundant at High (85% ERH) conditions (1 species)**		
Aspergillus penicillioides	<.0001	0.0001
**More abundant at saturated (100% ERH) conditions (8 species)**		
Penicillium chrysogenum	<.0001	<.0001
Penicillium gladioli	<.0001	<.0001
Aspergillus flavus	<.0001	0.0009
Aspergillus tamarii	0.0004	0.0079
Aspergillus nidulans	0.0008	0.0107
Aspergillus subversicolor	0.0007	0.0107
Penicillium concentricum	0.0008	0.0107
Aspergillus puniceus	0.0025	0.0296

Supplemental Table S22: Differential abundance fungal comparison between high (85% RH) for all TOW samples and all TOW saturated (100% RH) conditions. There were 30 fungal species more abundant in the high condition compared to 4 species more abundant at saturated conditions.

Fungal Species	Unadjusted P-value	Adjusted FDR P-value

**More abundant at high (85% ERH) conditions All TOW (30 species)**		
Alternaria alternata	<.0001	0.0004
Malassezia restricta	<.0001	0.0008
Epicoccum nigrum	<.0001	0.0008
Mycosphaerella tassiana	<.0001	0.0009
Gibberella intricans	0.0001	0.0014
Aspergillus penicillioides	0.0001	0.0014
Cladosporium delicatulum	0.0001	0.0014
Aspergillus conicus	0.0002	0.0018
Candida albicans	0.0003	0.0025
Malassezia globosa	0.0005	0.0039
Debaryomyces hansenii	0.0006	0.0039
Penicillium sclerotigenum	0.0007	0.0039
Colletotrichum gloeosporioides	0.0007	0.0039
Sarocladium implicatum	0.0007	0.0039
Phaeosphaeria podocarpi	0.0007	0.0039
Fusarium acutatum	0.0011	0.0056
Alternaria brassicae	0.0013	0.0066
Cyberlindnera jadinii	0.002	0.0095
Malassezia cuniculi	0.0029	0.0131
Penicillium aurantiogriseum	0.0034	0.015
Plectosphaerella oratosquillae	0.0059	0.0251
Pseudopithomyces chartarum	0.0063	0.0257
Fusarium culmorum	0.0075	0.0271
Penicillium citrinum	0.0077	0.0271
Penicillium thymicola	0.0071	0.0271
Zygosaccharomyces rouxii	0.0072	0.0271
Papiliotrema laurentii	0.011	0.0363
Saccharomyces cerevisiae	0.0132	0.0424
Verticillium dahliae	0.0152	0.0468
Lasiodiplodia brasiliensis	0.0155	0.0468
**More abundant at saturated Conditions (100% ERH) All TOW (4 species)**		
Aspergillus sydowii	<.0001	0.0013
Aspergillus nidulans	<.0001	0.0002
Aspergillus subversicolor	<.0001	0.0002
Aspergillus unguis	0.0005	0.0039

Supplemental Table 23: Example of bacterial differential abundance analysis for non-elevated (original dust and 50% RH) and elevated (80, 85, 90, and 100% RH) conditions for 2-week incubations. No bacterial species were found to be more abundant in either condition. This was true for all time-of-wetness incubation comparisons as well (not shown).

Bacterial Species	Unadjusted P-value	Adjusted FDR Pvalue
Corynebacterium kroppenstedtii	0.4687	0.5723
Staphylococcus pettenkoferi	0.4587	0.5723
Lactobacillus helveticus	0.4267	0.5723
Veillonella parvula	0.4783	0.5723
Acinetobacter rhizosphaerae	0.4827	0.5723
Veillonella dispar	0.4783	0.5723
Haemophilus parainfluenzae	0.4773	0.5723
Rothia mucilaginosa	0.4818	0.5723
Faecalibacterium prausnitzii	0.4799	0.5723
Corynebacterium stationis	0.3218	0.5723
Rothia dentocariosa	0.4833	0.5723
Streptococcus infantis	0.4765	0.5723
Staphylococcus saprophyticus	0.2376	0.5723
Corynebacterium variabile	0.1595	0.5723
Streptococcus anginosus	0.4834	0.5723
Corynebacterium durum	0.4789	0.5723
Capnocytophaga ochracea	0.488	0.5723
Prevotella melaninogenica	0.4826	0.5723
Roseburia faecis	0.4532	0.5723
Lachnoanaerobaculum orale	0.4901	0.5723
Gemmiger formicilis	0.4199	0.5723
Rothia aeria	0.4932	0.5723
Aggregatibacter segnis	0.486	0.5723
Sphingobium yanoikuyae	0.3243	0.5723
Campylobacter ureolyticus	0.4823	0.5723
Brevibacterium paucivorans	0.5421	0.5834
Pseudomonas veronii	0.2718	0.5723
Staphylococcus succinus	0.3254	0.5723
Blautia obeum	0.2901	0.5723
Clostridium clostridioforme	0.4763	0.5723
Prevotella nanceiensis	0.4921	0.5723
Ruminococcus bromii	0.7364	0.7422
Bacteroides uniformis	0.4816	0.5723
Actinomyces europaeus	0.4813	0.5723
Neisseria subflava	0.4414	0.5723
Staphylococcus equorum	0.4831	0.5723
Selenomonas noxia	0.4827	0.5723

Micrococcus luteus	0.3841	0.5723
Kocuria palustris	0.4722	0.5723
Prevotella copri	0.3542	0.5723
Coprococcus eutactus	0.4756	0.5723
Neisseria oralis	0.5037	0.5723
Staphylococcus aureus	0.4904	0.5723
Lactobacillus delbrueckii	0.4541	0.5723
Prevotella intermedia	0.597	0.6266
Campylobacter rectus	0.5022	0.5723
Porphyromonas endodontalis	0.2417	0.5723
Actinobacillus porcinus	0.0523	0.5723
Xanthomonas campestris	0.4035	0.5723
Acinetobacter guillouiae	0.1078	0.5723
Lactobacillus iners	0.4833	0.5723
Bacteroides caccae	0.4824	0.5723
Prevotella pallens	0.5112	0.5723
Raphanus sativus	0.3651	0.5723
Ruminococcus callidus	0.6408	0.6563
Brevibacterium aureum	0.3173	0.5723
Dorea formicigenerans	0.4045	0.5723
Malus domestica	0.2903	0.5723
Sphingomonas yabuuchiae	0.5137	0.5723
Lactobacillus hamsteri	0.5267	0.5767
Bacteroides ovatus	0.061	0.5723
Alistipes putredinis	0.4847	0.5723
Abiotrophia defectiva	0.0553	0.5723
Bulleidia moorei	0.4814	0.5723
Prevotella nigrescens	0.4868	0.5723
Actinobacillus parahaemolyticus	0.5525	0.5893
Coprococcus catus	0.5251	0.5767
Neisseria cinerea	0.1916	0.5723
Roseburia inulinivorans	0.0275	0.5723
Lactobacillus zeae	0.3927	0.5723
Bifidobacterium adolescentis	0.5381	0.5834
Acinetobacter lwoffii	0.4828	0.5723
Blautia producta	0.6528	0.6632
Collinsella aerofaciens	0.3588	0.5723
Haemophilus influenzae	0.0035	0.4473
Dorea longicatena	0.5568	0.5893

Akkermansia muciniphila	0.4866	0.5723
Eubacterium biforme	0.6081	0.6279
Ruminococcus gnavus	0.4971	0.5723
Tetragenococcus halophilus	0.415	0.5723
Ruminococcus lactaris	0.6055	0.6279
Prevotella tannerae	0.1506	0.5723
Propionibacterium acnes	0.7683	0.7683
Parabacteroides distasonis	0.4786	0.5723
Staphylococcus haemolyticus	0.5056	0.5723
Staphylococcus lugdunensis	0.0714	0.5723
Alistipes onderdonkii	0.0174	0.5723
Clostridium celatum	0.4807	0.5723
Streptococcus luteciae	0.4011	0.5723
Bacteroides plebeius	0.4228	0.5723
Butyricicoccus pullicaecorum	0.3384	0.5723
Atopobium rimae	0.464	0.5723
Corynebacterium aurimucosum	0.3477	0.5723
Rothia nasimurium	0.2269	0.5723
Jeotgalicoccus psychrophilus	0.3406	0.5723
Ruminococcus torques	0.233	0.5723
Cardiobacterium valvarum	0.4312	0.5723
Geobacillus vulcani	0.3282	0.5723
Paracoccus aminovorans	0.3156	0.5723
Psychrobacter marincola	0.305	0.5723
Corynebacterium simulans	0.4117	0.5723
Ruminococcus albus	0.4256	0.5723
Enhydrobacter aerosaccus	0.3782	0.5723
Brevibacterium casei	0.3777	0.5723
Alistipes finegoldii	0.3198	0.5723
Anoxybacillus kestanbolensis	0.3445	0.5723
Staphylococcus sciuri	0.3351	0.5723
Neisseria bacilliformis	0.463	0.5723
Bacteroides coprophilus	0.3823	0.5723
Psychrobacter pulmonis	0.3095	0.5723
Flavobacterium succinicans	0.2183	0.5723
Arabidopsis thaliana	0.0851	0.5723
Rothia amarae	0.1633	0.5723
Peptostreptococcus anaerobius	0.3786	0.5723
Pseudoclavibacter bifida	0.2885	0.5723
Erwinia chrysanthemi	0.2708	0.5723

Bacillus coagulans	0.2688	0.5723
Cucurbita pepo	0.4661	0.5723
Treponema socranskii	0.4393	0.5723
Methylobacterium adhaesivum	0.3441	0.5723
Streptococcus agalactiae	0.4272	0.5723
Paracoccus marcusii	0.3902	0.5723
Acinetobacter schindleri	0.3701	0.5723
Micrococcus terreus	0.4271	0.5723
Enterococcus cecorum	0.4012	0.5723
Cupriavidus gilardii	0.2283	0.5723
Alloiococcus otitis	0.4274	0.5723

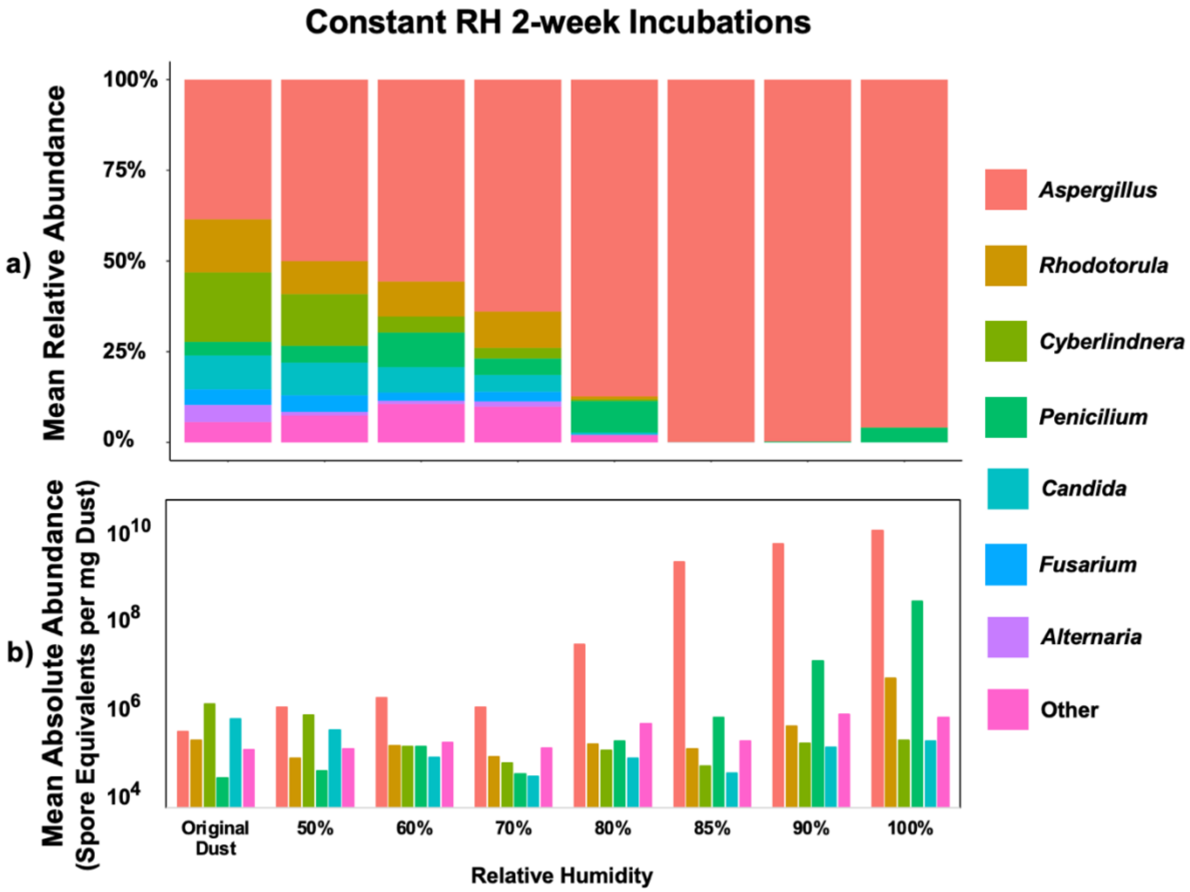

Supplemental Figure S8: Mean relative (A) and absolute (B) abundance data for fungal genus in constant equilibrium relative humidity (ERH) incubation samples. Original dust was not incubated and represents what was in the dust in the ISS vacuum bags with no ERH exposure. For each ERH condition, samples were incubated for 2 weeks at 25°C. Each condition (including original dust) represents the mean of 12 total dust samples which includes 3 physical triplicates from the 4 ISS vacuum bags used in this study.

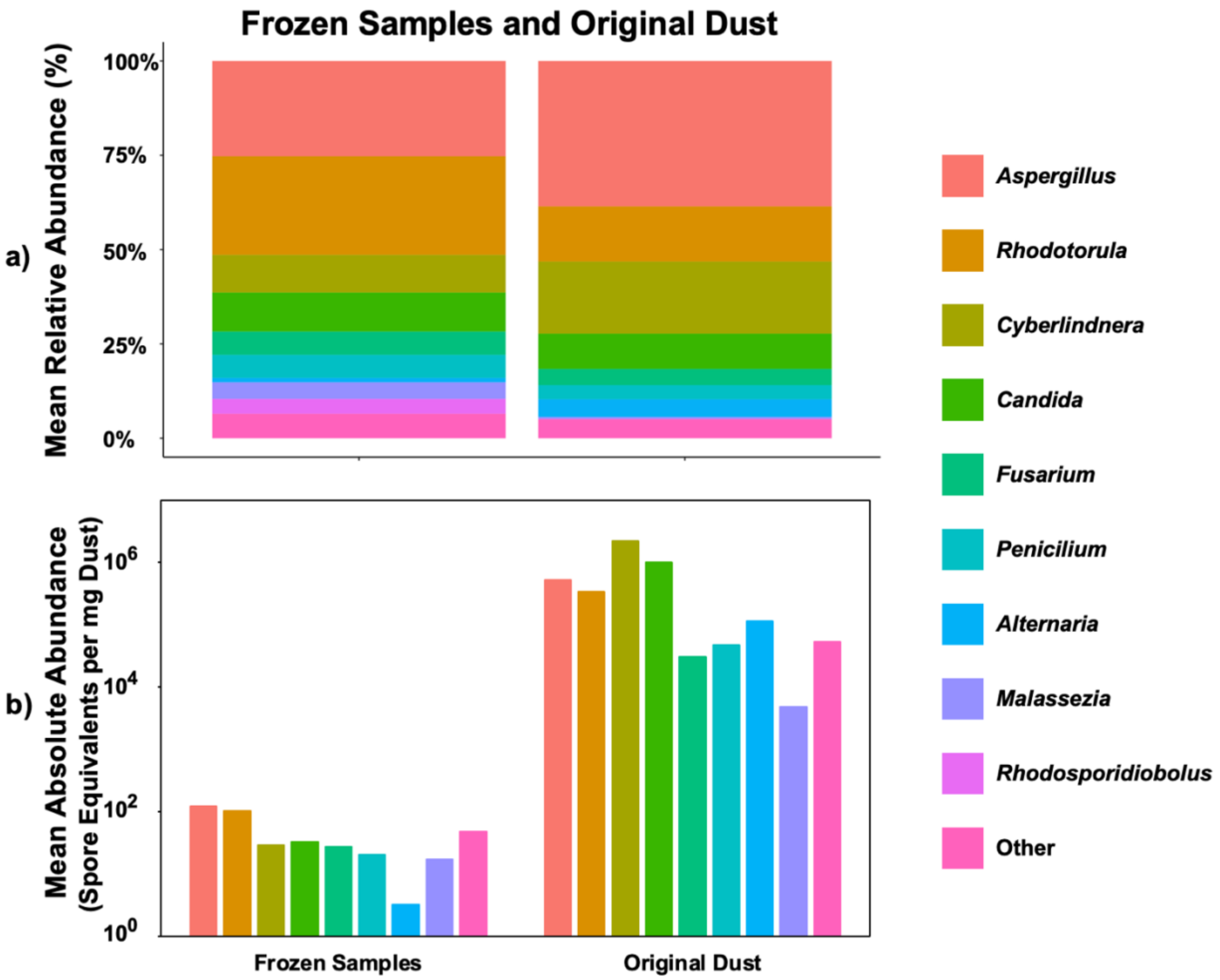

Supplemental Figure S9: Mean relative (A) and absolute (B) abundance data for fungal genus of frozen ISS dust and original dust samples. Original dust was not incubated and represents what was in the dust in the ISS vacuum bags with no ERH exposure. Frozen dust samples were collected onboard the ISS via a tweezer (no vacuum), placed in a triple-sealed plastic bag, and stored at -80°C until use in this study. The abundance data represents of a total of 12 samples for original dust samples (3 for each ISS bag) and 8 frozen dust samples (1 for each location sampled).
